# Effect of Ethylene on Cell Wall and Lipid Metabolism during Alleviation of Postharvest Chilling Injury in Peach

**DOI:** 10.3390/cells8121612

**Published:** 2019-12-11

**Authors:** Yongchao Zhu, Ke Wang, Chunxia Wu, Yun Zhao, Xueren Yin, Bo Zhang, Don Grierson, Kunsong Chen, Changjie Xu

**Affiliations:** 1 College of Agriculture & Biotechnology, Zhejiang University, Zijingang Campus, Hangzhou 310058, China21716147@zju.edu.cn (C.W.); donald.grierson@nottingham.ac.uk (D.G.); akun@zju.edu.cn (K.C.); 2 Anhui Engineering Laboratory for Agro-products Processing, Anhui Agricultural University, Hefei 230036, China; wangke@ahau.edu.cn; 3 Zhejiang Provincial Key Laboratory of Horticultural Plant Integrative Biology, Zhejiang University, Zijingang Campus, Hangzhou 310058, China; 11416049@zju.edu.cn (Y.Z.); xuerenyin@zju.edu.cn (X.Y.); bozhang@zju.edu.cn (B.Z.); 4 Plant Sciences Division, School of Biosciences, University of Nottingham, Sutton Bonington Campus, Loughborough LE12 5RD, UK

**Keywords:** ABR1, cell wall, ERFs, ESE3, ethylene, lipid, peach, postharvest chilling injury

## Abstract

Peach is prone to postharvest chilling injury (CI). Here it was found that exogenous ethylene alleviated CI, accompanied by an increased endogenous ethylene production. Ethylene treatment resulted in a moderately more rapid flesh softening as a result of stronger expression of genes encoding expansin and cell wall hydrolases, especially xylosidase and galactosidase. Ethylene treatment alleviated internal browning, accompanied by changes in expression of *polyphenol oxidase*, *peroxidase* and *lipoxygenases*. An enhanced content of phospholipids and glycerolipids and a reduced content of ceramide were observed in ethylene-treated fruit, and these were associated with up-regulation of *lipid phosphate phosphatase*, *fatty acid alpha-hydroxylase*, and *golgi-localized nucleotide sugar transporter*, as well as down-regulation of *aminoalcohol phosphotransferases*. Expression of two *ethylene response factors* (*ERFs*), *ESE3* and *ABR1*, was highly correlated with that of genes involved in cell wall metabolism and lipid metabolism, respectively. Furthermore, the expression of these two *ERFs* was strongly regulated by ethylene treatment and the temperature changes during transfer of fruit into or out of cold storage. It is proposed that ERFs fulfill roles as crucial integrators between cell wall modifications and lipid metabolism involved in CI processes ameliorated by exogenous ethylene.

## 1. Introduction

Peach can develop chilling injury (CI) during cold storage or after transfer to the shelf when the fruit were stored for long periods [1,2]. The most common symptoms of CI in peach are internal browning (IB) of flesh and impairment of softening [3].

IB is thought to be associated with the low temperature-induced damage of cellular membranes, allowing the enzymatic oxidation of phenolic compounds catalyzed by polyphenol oxidase (PPO) located in the cytoplasm [4,5], generating brown *o*-quinones which lead to the occurrence of browning tissue [6]. It is generally accepted that the browning of fruit under low temperature is often associated with damage to membrane integrity [7,8]. Changes in lipid content and unsaturation affect the integrity and mobility of cell membranes and a higher unsaturation of membrane lipids leads to an enhanced tolerance to chilling stress by maintaining membrane fluidity in peach [8]. Benefiting from the breakthrough of lipid analysis technology, the role and mechanism of lipid metabolism changes in plant cold stress responses has recently been investigated. The changes of lipid composition and key related genes that respond to low temperature stress have been identified, indicating that phospholipid and sphingolipid (especially ceramide, Cer) metabolism play important roles in plant tolerance to cold stress [9,10,11,12].

Softening is one of the main physiological characteristics occurring during ripening of fleshy fruits. However, low temperature can induce CI symptoms characterized by the loss of ability to soften normally [13] and this softening impairment has been attributed to the change in activity of cell wall metabolism enzymes [3,14]. Expressions of *polygalacturonase* (*PG*), *pectate lyase* (*PLY*) and *expansin* (*Exp*) increase during fruit softening [15,16]. *Exp* as well as cell wall hydrolase genes *PLY*, *xylosidase* (*Xyl*), *β-1, 4-endoglucanase* (*EGase*) and *xyloglucan endo-transglucosylase/hydrolase* (*XTH2*) displayed lower transcript abundance in fruit with CI [1,17].

Ethylene is a key factor determining fruit ripening, especially climacteric ones, and plays an important role in regulating responses of plants to cold stress. In whole plant, evidences from genetics, biochemistry, and molecular biology have shown that ethylene can have either a positive or a negative effect on cold stress symptoms, depending on the plant species studied. On the one hand, ethylene has been reported to reduce cold tolerance by repressing expression of *CBF* and type-A *Arabidopsis response regulator* (*ARR*) gene in *Arabidopsis* [18]. On the other hand, increased cold tolerance induced by ethylene was found in grapevine, tobacco, and tomato plants [19,20]. In postharvest fruits, ethylene or its response inhibitor 1-methylcyclopropene (1-MCP) have been reported to affect CI occurrence as well. CI symptoms of some fruits, such as banana [21], papaya, [22] and pear [23] can be alleviated by exogenous ethylene. Similarly, increased CI of tomato fruit was observed following application of ethylene response inhibitor 1-MCP [24]. However, in peach fruit, the effect of ethylene on CI appears to be in dispute. Ethylene-alleviated and 1-MCP-induced CI has been reported in nectarines [25,26,27], which contrasted with some other reports where alleviation of CI was achieved by blocking ethylene action with 1-MCP [28,29]. On investigation of the mechanisms for alleviation of postharvest fruit CI in peach by low temperature conditioning (LTC), we previously found that the alleviation was related to a significantly enhanced ethylene biosynthesis during early stage of cold storage [1]. In this study, we aimed to clarify whether exogenous application of ethylene can alleviate the CI of peach fruit, and to understand the similarities and differences in the alleviation mechanisms between LTC and ethylene.

Previous studies have shown that ethylene receptors, ethylene signaling transduction elements and ethylene response factors (ERFs) are involved in ethylene response to low temperature [30,31,32]. Some ERF members have been shown to regulate cold tolerance in plants, such as TERF2 [33], JERF3 [34], VaERF057 [19], and GmERF9 [35]. In peach, a number of cold-related ERF members, such as CBF2/4 and ERF1/25/61/118, have been identified in our previous study [1]. Some ERFs, such as AtWRI1/3/4, have been found to directly or indirectly regulate lipid genes in *Arabidopsis* [36,37]. However, reports on ERF regulation of lipid genes have focused mainly on *Arabidopsis* [36,38,39,40], and the investigation of the regulation of lipid genes by ERFs have rarely been reported in fruits.

To date, the effect of ethylene on CI of fruits is still controversial and the underlying mechanisms remain elusive. Here, we undertook a transcriptomic and lipidomics approach to investigate whether ethylene has a positive role in the alleviation of CI of peach and to identify *ERFs*, cell wall and lipid metabolism genes with expression changed in response to ethylene, as well as to analyze the intrinsic relationships between them. 

## 2. Materials and Methods

### 2.1. Plant Material and Treatments

Fruit of a non-melting peach (*Prunus persica* Batsch) variety, “Zhonghuashoutao”, were transported from a commercial orchard, Linyi, Shandong, China to the lab on the day of harvest. Fruits of uniform size, around 7–8 cm in diameter, were selected and randomly divided into two groups. For one group, the fruit were constantly treated with ethylene (100 μL L^−1^) in air for 24 h at 20 °C in a 20 L plastic container followed by 28 d at 5 °C. The fruit in the other group were sealed in a 20 L plastic container containing air and served as the control. During 5 °C storage, the containers were opened, ventilated and ethylene treatment or control re-established every 24 h. After storage at 5 °C for 28 d, the fruit were transferred to 20 °C for 2 d for subsequent ripening. For clarity, these 2 d are indicated as ‘+2′. Fruit were sampled at 0, 7, 14, 28 d during cold storage as well as at +2 d of subsequent ripening following 28 d cold storage. For each sampling point, 3 replicates of 4 fruits were collected from each treatment. Mesocarp samples were sliced, frozen in liquid nitrogen, and stored at −80 °C for further experiments.

### 2.2. Measurements of Ethylene, Firmness, and Browning

Ethylene analysis was performed according to the method described previously [1]. Fruit were sealed in a plastic jar for 1 h at 5 °C when fruit were in cold storage or at 20 °C when the fruit were on shelf, and 1 mL gas sample was taken to determine ethylene by gas chromatography (Agilent Technologies 7890A GC System, Santa Clara, CA, USA). Firmness was measured on opposite sides at the equator of each fruit after removal of 1 mm-thick slice of skin with a texture analyzer (TA-XT2i Plus; Stable Micro System Ltd., Surrey, UK) with a 7.5 mm-diameter head, following the method described previously [1]. An IB index was used to evaluate the degree of internal browning based on our previous study [1], and was calculated on the basis of IB rating with the following formula: IB index = 100% × Σ (internal browning scale) × (number of fruit at that internal browning scale) / (4 × total number of fruit evaluated). Ethylene production was expressed as μL h^−1^ kg^−1^ fresh weight (FW). Firmness was expressed as Newton (N).

### 2.3. Measurements of Fatty Acids 

Fatty acid determination was performed as described previously [1]. In brief, a solution of hexane: isopropanol (3:2, *v*/*v*) was used to extract fatty acids. Heptadecanoic acid (C17:0; Sigma-Aldrich Corp., Saint Louis, MO, USA) was added to serve as an internal standard. Methanol: toluene: H_2_SO_4_ (88:10:2) was added to the residue at 80 °C for methyl esterification. Anhydrous Na_2_SO_4_ was added to remove residual water and then the upper phase used for fatty acid measurement. The analysis was carried out with a gas chromatograph (Agilent 6890N) equipped with a DB-23 column (0.25 mm, 30 m, 0.25 μm, J&W Scientific, Folsom, CA, USA), and helium was used as the carrier gas with a flow rate of 1.2 mL min^−1^. Conditions for chromatography were as follows: injection, 250 °C; initial oven temperature, 50 °C, increased to 200 °C by 25 °C min^−1^, increased to 230 °C by 3 °C min^−1^, and held for 5 min. Identification and quantitative determination of compounds was carried out using the peak of the internal standard as a reference value and calculated by comparing with authentic standards. Fatty acid content was expressed as μg g^−1^ fresh weight (FW).

### 2.4. Transcriptome Sequencing and Data Analysis

RNA was extracted by the method described by Meisel et al. [41]. RNA concentration was measured using NanoDrop 2000 (Thermo Fisher Scientific Inc., Waltham, MA, USA). RNA integrity was assessed using the RNA Nano 6000 Assay Kit of the Agilent Bioanalyzer 2100 system (Agilent Technologies, Santa Clara, CA, USA). RNA-seq analysis was performed at Biomarker Technologies Co. Ltd. (Beijing, China) on an Illumina platform and paired-end reads were generated. Raw data (raw reads) of fastq format were first processed through in-house perl scripts. In this step, clean data (clean reads) were obtained by removing reads containing adapter, reads containing poly-N and low-quality reads from raw data. At the same time, Q30, GC-content and sequence duplication level of the clean data were calculated. All the downstream analyses were based on clean data. 

The index of the reference genome obtained from the genome database (GDR, http://www.rosaceae.org/species/prunus_persica/genome) was built using Bowtie v2.0.6. [42]. Hisat2 tools soft [43] were used to map with the reference genome. HTSeq v0.5.4p3 [44] was used to count the read numbers mapped to each gene. Quantification of gene expression levels was estimated by fragments per kilobase of transcript per million fragments (FPKM) mapped. Differential expression analysis of two groups was performed using the DESeq R package (1.10.1) [45]. The resulting *p*-values were adjusted using the Benjamini and Hochberg’s approach for controlling the false discovery rate (FDR). Genes with a *p*-value < 0.05 found by DESeq were assigned as differentially expressed (DEGs). DEGs with at least 2-fold change for at least one time point between ethylene treatment and the control were selected for further analysis.

### 2.5. Weighted Gene Coexpression Network Analysis 

Weighted gene coexpression network analysis (WGCNA) on DEGs were performed using R package [46]. United DEGs (4350, *p* < 0.05, log_2_FoldChange >1 for at least one time point) in all comparisons of samples were used to conduct coexpression analysis to identify ethylene regulatory modules. A pairwise Pearson correlation matrix was created for all genes and further transformed into a weighted matrix with a thresholding power of seven. The topological overlap metric (TOM) was derived from the resulting adjacency matrix [47]. This resulted in a cluster dendrogram which was used for module detection by using the dynamic tree cut method (TOMthype = “unsigned”, minModuleSize = 30, mergeCutHeight = 0.25). Intramodular connectivity based on module eigengenes was used to identify hubs in the modules. In order to further explore the association of modules to external traits, the eigengenes of each module were correlated to physiological and biochemical indexes using a Spearman rank correlation. Cytoscape 3.6.1 software [48]. was used to display a coexpression network of genes putatively interacting in each module.

### 2.6. Lipidome Analysis

Lipidome analysis was performed by staff at Shanghai Applied Protein Technology Co. Ltd. (Shanghai, China). Lipid extraction was performed according to a previously reported method [49]. In brief, 40 mg frozen sample was ground to powder in liquid nitrogen, and then homogenized with the solution including 200 μL distilled water, 240 μL pre-cooling methanol, and 800 μL methyl tert-butyl ether. The total lipid extract was dried under a gentle stream of nitrogen. The sample was dissolved in 100 μL isopropanol and analyzed with an ultra-high-performance liquid chromatography (UHPLC) Nexera LC-30A C18 column (100 mm × 2.1 mm, 1.7 μm) at 45 °C. Mobile phases were as follows: phase A, acetonitrile: water (6:4, *v*/*v*) containing 10 mM ammonium formate; phase B, acetonitrile: isopropanol (1:9, *v*/*v*) containing 10 mM ammonium formate and 0.1% formic acid. The liquid chromatography elution gradients were as follows: 0–2 min, 70% A and 30% B; 2–25 min, solvent B was linearly increased from 30% to 70% and solvent A was linearly decreased from 70% to 30%; 25–30 min, the column was equilibrated with 95% solvent A and 5% solvent B. The flow rate was 0.4 mL min^−1^ and the injection volume was 4 μL.

Mass spectrometry (MS) was recorded under both positive and negative electrospray ionization (ESI) modes. ESI conditions were as follow: heater temperature 300 °C, sheath gas flow rate 45 arb, auxiliary gas flow rate 15 arb, sweep gas flow rate 1 arb, spray voltage 3.0 KV (positive mode) and 2.5 KV (negative mode), capillary temperature 350 °C, S-Lens radio frequency (RF) level 50% (positive mode) and 60% (negative mode) and scan ranges *m*/*z* 200–1800 (positive mode) and *m*/*z* 250–1800 (negative mode). Scan mode of full MS (resolution 70,000) and ddms3 (resolution 17,500; CID 35) were applied for both positive and negative modes.

The raw data for all samples were identified by the LipidSearch software version 4.1 (Thermo Fisher Scientific Inc., Waltham, MA, USA). Fold change of the lipid content between ethylene and control was calculated and Student’s test was used to compare two sets (*n* = 4) at the same point. The difference between ethylene and control was interpreted as being significant if *p*-value < 0.05.

### 2.7. Real-Time Quantitative PCR 

Real-time quantitative PCR (qPCR) was performed with SsoFast EvaGreen Supermix Kit (Bio-Rad, Hercules, CA, USA) using a CFX96 instrument (Bio-Rad, Hercules, CA, USA). TransCript All-in-One First-Strand cDNA Synthesis SuperMix (TransGen Biotech, Beijing, China) for qPCR was used to synthesize cDNA. The primers used for qPCR are listed in Table S1. The reactions were performed as follows: denaturation at 95 °C for 5 min, 45 cycles at 95 °C for 10 s, 60 °C for 30 s, and then 95°C for 10 s followed by a continuous increase from 65 °C to 95 °C with a ramp rate of 0.5 °C s^−1^ for dissociation curve analysis. Peach gene translation elongation factor 2 (*PpTEF2*, Prupe. 4G138700) was used as reference gene to normalize all target gene expressions. 

### 2.8. Statistical Analysis

Statistical analysis was performed with SPSS 17.0 (SPSS Inc., Chicago, IL, USA). A significantly higher level in one treatment was defined as at least two time points with significantly higher level and no time point showing a significantly lower level, and allowing the presence of time points with no significant difference. A *p*-value < 0.05 was considered statistically significant.

## 3. Results

### 3.1. Ethylene Alleviates IB and Promotes Softening in Peach Fruit

**Figure 1:**
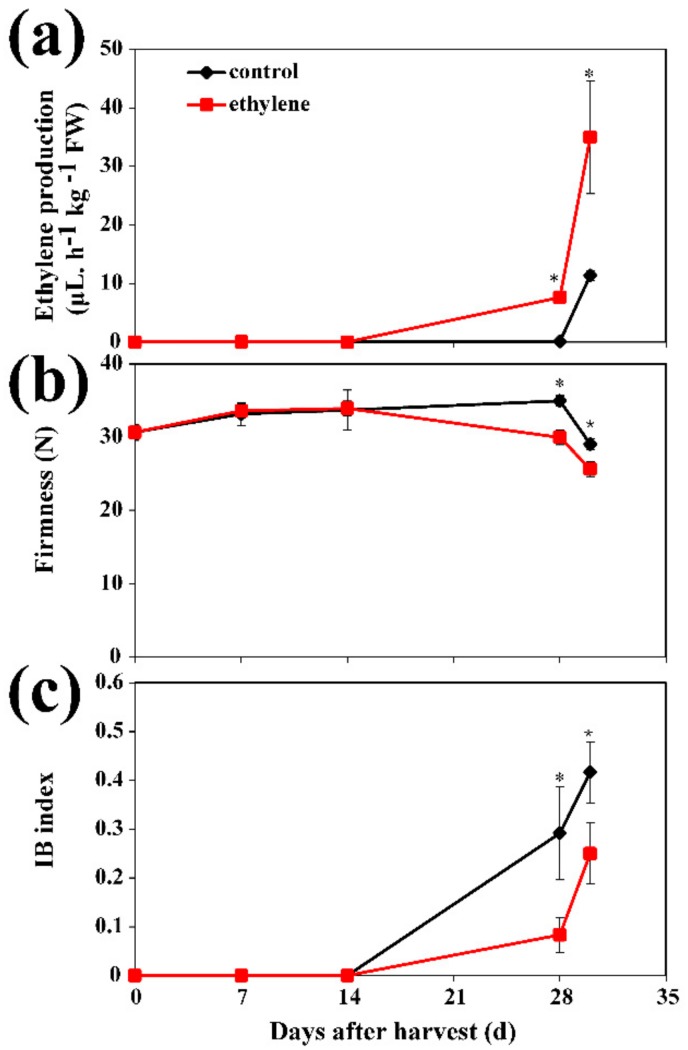
Ethylene-induced physiological changes in peach fruit. (**a**) The profile of ethylene production rate; (**b**) flesh firmness; (**c**) internal browning (IB) index. Fruit were transferred to 20 °C for 2 d after 28 d of cold storage (28 + 2). Data are presented as mean ±SE from three independent biological replicates. Asterisks (*) indicate that mean values are significantly different between ethylene treatment and control (*p* < 0.05) according to Duncan’s multiple range test.

The effect of exogenous ethylene on postharvest CI of ‘Zhonghuashoutao’ peach was investigated. Ethylene production remained at a lower level during cold storage and increased to a moderate level during 2 d on the shelf, indicated as +2 d, at 20 °C. Ethylene levels of control fruit showed a lower value during cold storage and during the subsequent +2 d on the shelf, while a significantly higher level of ethylene production was observed in fruit treated with exogenous ethylene during 28 d of cold storage and +2 d of shelf storage (Figure 1a). There was no significant difference in the firmness of peaches between the control and ethylene-treated fruit at or before 14 d in cold storage. A moderately lower firmness was observed in ethylene-treated fruit at 28 d in cold storage and +2 d on shelf (Figure 1b). IB was not observed in any fruit during the first 14 d of storage, while it was obvious at 28 d in cold storage and +2 d of shelf. IB index was significantly lower in ethylene-treated fruit compared with that in control fruit (Figure 1c). These data showed the positive role of ethylene on alleviation of postharvest CI in peach.

### 3.2. Overview of RNA-Seq Analysis

To explore the molecular basis for alleviation of CI by ethylene, RNA-Seq analysis was conducted to generate transcriptome profiles on the samples described above with three biological replicates for each treatment. In total, 27 libraries were constructed and analyzed. The data were deposited in the NCBI SRA database (SRA accession number: SRP219124). The number of clean reads for each library was over 20 million with Q30 over 94% (Table S2). The RNA-Seq reads were mapped to the peach genome with uniquely mapped reads reaching around 90% (Table S3). Pearson correlation analysis indicated that all libraries from the biological replicates showed high correlation coefficients (Table S4), and hence the quality of RNA-Seq analysis was high enough to support the subsequent analysis.

### 3.3. Differential Gene Expression Analysis

**Figure 2:**
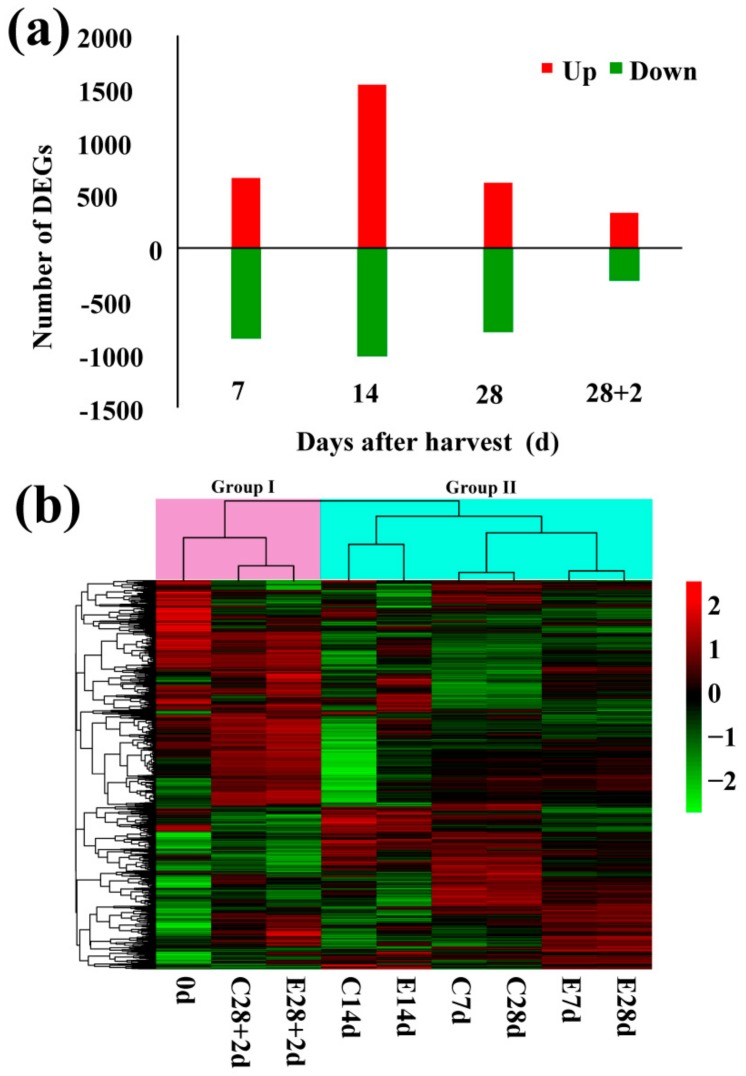
Overview of transcriptome of peach fruit at given time points during the ethylene treatments. (**a**) Number of differentially expressed genes (DEGs) identified by pairwise comparison between ethylene treatment and control at each time point (*p* < 0.05). Up, up-regulation; Down, down-regulation. (**b**) Hierarchy clustering of DEGs (*p* < 0.05, and log_2_foldchange > 1) across the different samples. The rows in the heatmap represent genes, and the columns indicate samples. The colors of heatmap cells indicate scaled expression level of genes (log_2_ FPKM) across different samples. The color gradient, ranging from green, through black, to red represents low, middle and high values of gene expression. The sampling points beginning with the letter C or E indicate those belong to control and ethylene treatments, respectively. FPKM, fragments per kilobase of transcript per million fragments mapped.

To investigate the transcript differences between control and ethylene-treated fruit, specifically expressed genes in each sampling point were identified. Expression levels were measured as FPKM. Log_2_Foldchange >1, *p* < 0.05 were used as the threshold to assess significant difference in gene expression. A total of 4350 DEGs were identified between ethylene treatment and control, with 2450 and 1900 genes exhibiting significantly higher or lower expression, respectively. Interestingly, both the number of DEGs up-regulated or down-regulated by ethylene was highest at day 14, and then gradually declined up to +2 d shelf storage (Figure 2a). Hierarchical cluster analysis (HCA) sorted the samples into two groups, with Group I containing fruit sampled at 20 °C on the left part of the panel and Group II containing fruit sampled at 5 °C. In Group II, fruit sampled at 7 d and 28 d were clustered into a subgroup with those at 14 d in another subgroup (Figure 2b). E28d fruit were mostly closed to E7d rather than to C28d (Figure 2b), suggesting the stronger effects of ethylene treatment over storage duration. The two- and three-dimensional principal component analysis revealed that control and ethylene-treated samples were well separated (Figure S1).

**Figure 3:**
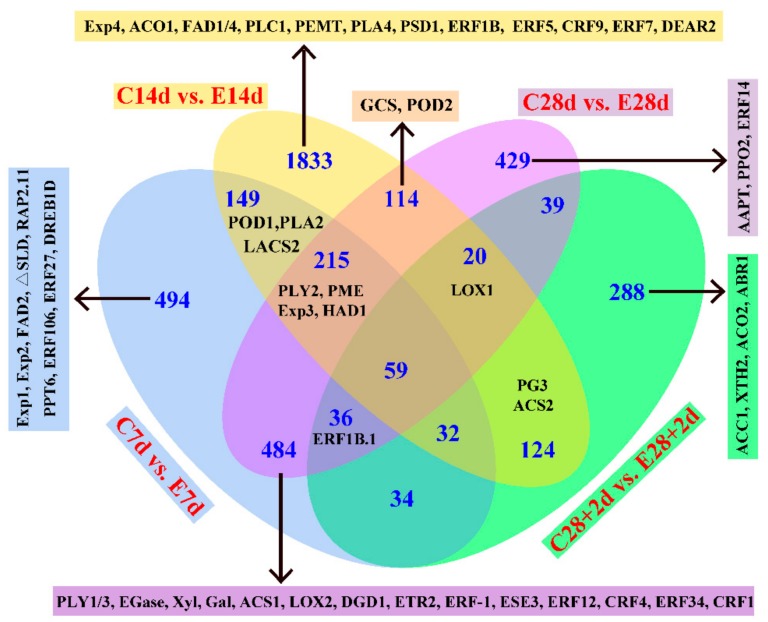
Venn diagrams illustrating the number of differentially expressed genes (DEGs) revealed by paired comparison between ethylene treated and control peach fruit. AAPT, aminoalcoholphosphotransferase; ACO, 1-aminocyclopropane-1-carboxylic acid (ACC) oxidase; ACS, ACC synthase; EGase, β-1, 4-endoglucanase; ETR, ethylene receptor; Exp, Expansin; FAD, fatty acid desaturase; Gal, galactosidase; GCS, glucosylceramide synthase; LACS, long-chain acyl-CoA synthetase; LOX, lipoxygenase; PG, polygalacturonase; PLC, phospholipase C; PLY, pectate lyase; PME, pectin methylesterase; POD, peroxidase; PPO, polyphenol oxidase; Xyl, xylosidase; XTH, xyloglucan endo-transglucosylase/hydrolase. The sampling points beginning with letter C or E indicate those belong to control and ethylene treatments, respectively.

Overall expression patterns of DEGs were illustrated with Venn diagrams presented in Figure 3. Gene Ontology (GO) analysis showed that DEGs were mainly involved in metabolic process, response to stimulus, biological regulation, and signaling (Figure S2). Of the 4350 DEGs, 59 DEGs were significantly differentially expressed in the two pairwise comparisons between ethylene and control at all four time points (Figure 3). A total of 13 cell wall metabolism related genes, 16 lipid genes, 5 browning genes, 5 ethylene biosynthesis or signaling element genes and 19 *ERFs* genes were found to be differentially expressed between ethylene-treated fruit and the control (Figure 3). 

qPCR analyses were performed for critical genes generally regarded in plants as being related to lipid, ethylene and cell wall metabolism [1]. The correlation coefficient between the RNA-seq and qPCR was over 0.8 for all genes (Figure S3). The results indicated that the expression data from RNA-seq analysis well matched those from qPCR and therefore are reliable.

### 3.4. Expression of Genes Related to Ethylene Biosynthesis and Signaling Pathway

**Figure 4:**
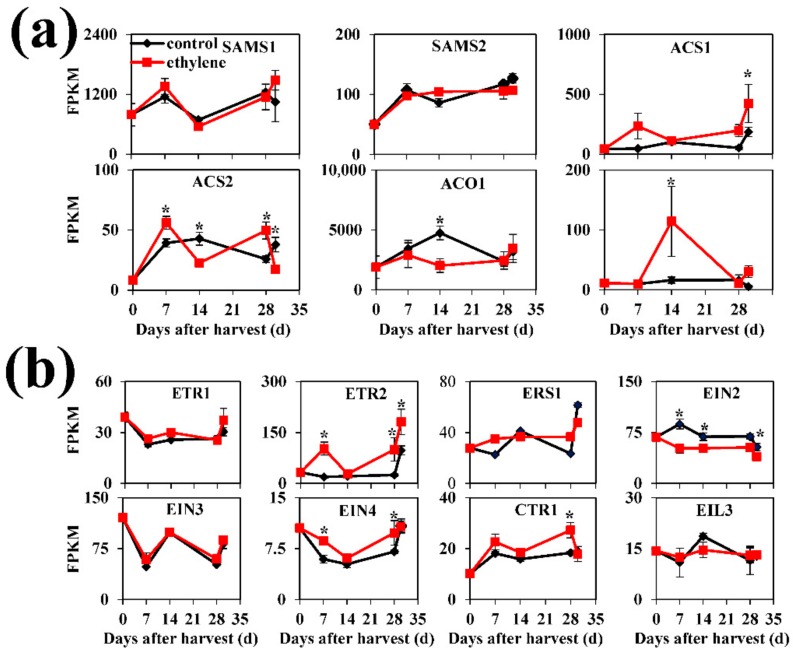
Expression profiles of genes related to ethylene biosynthesis (**a**) and ethylene signaling pathway (**b**) in peach fruit. Data are presented as mean ±SE from three independent biological replicates. Fruit were transferred to 20 °C for 2 d after 28 d of cold storage (28 + 2). Asterisks (*) indicate that mean values are significantly different between ethylene treatment and control (*p* < 0.05) according to Duncan’s Multiple Range Test. ACO, 1-aminocyclopropane-1-carboxylic acid (ACC) oxidase; ACS, ACC synthase; CTR, constitutive triple response; EIL, ethylene-insensitive3-like; EIN, ethylene insensitive; ESR, ethylene-sensitive-related; ETR, ethylene receptors; FPKM, fragments per kilobase of exon per million reads mapped; SAMS, S-adenosylmethionine synthase.

To understand the mechanisms for ethylene-induced endogenous ethylene production, the expression of genes involved in ethylene biosynthesis and signaling, with IDs listed in Table S5, was investigated. Transcript levels of *S-adenosylmethionine synthetase 1-2* (*SAMS1*-2), *1-aminocyclopropane-1-carboxylic acid (ACC) synthase 2* (*ACS2*), and *ACC oxidase 1* (*ACO1*) had no overall significant difference between control and ethylene treatment, while transcript levels of *ACS1* and *ACO2* were significantly induced by ethylene treatment (Figure 4a). Thus, our results indicated that exogenous ethylene promotes the increase in endogenous ethylene production by up-regulating specific ethylene biosynthesis-related genes in peach fruit during cold storage.

The expression of ethylene receptors and signaling elements were also evaluated to further understand the ethylene response. Transcript level of *ethylene receptor* (*ETR2*) increased during storage, and ethylene treatment significantly enhanced the expression of *ETR2* (Figure 4b). Moreover, ethylene induced the increase in *ethylene insensitive* (*EIN4*) transcript abundance at 14 and 28 d of cold storage. However, *EIN2*, another *EIN* member, exhibited a lower expression level in ethylene-treated fruit (Figure 4b). The upstream element of EIN2, *constitutive triple response 1* (*CTR1*) showed increased transcript level at 28 d of cold storage (Figure 4b). Therefore, the alleviation of ethylene on CI response was associated with decreased expression of *EIN2* and increased expression of downstream signaling elements *ETR2*, *CTR1* and *EIN4*.

### 3.5. Expression of Genes Related to Flesh Softening

**Figure 5:**
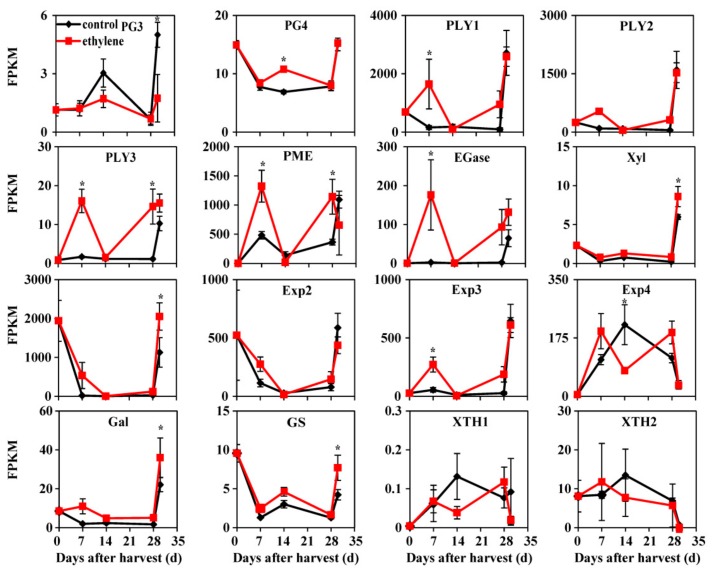
Expression profiles of genes related to cell wall metabolism. Data are presented as mean ±SE from three independent biological replicates. Fruit were transferred to 20 °C for 2 d after 28 d of cold storage (28 + 2). Asterisks (*) indicate that mean values are significantly different between ethylene treatment and control (*p* < 0.05) according to Duncan’s multiple range test. EGase, β-1, 4-endoglucanase; Exp, expansin; Gal, galactosidase; GS, α-1,4-glucan-protein synthase; PG, polygalacturonase; PLY, pectate lyase; PME, pectin methylesterase; Xyl, xylosidase; XTH, xyloglucan endo-transglucosylase/hydrolase.

Genes encoding enzymes involved in cell wall remodeling, with IDs listed in Table S5, generally showed higher transcript abundance in ethylene-treated fruit (Figure 5). *PLY3* and *pectin methylesterase* (*PME*) showed higher transcript levels in ethylene-treated fruit during cold storage. *Xyl*, *Exp1*, *galactosidase* (*Gal*), and *alpha-1,4-gluncan-protein synthase* (*GS*) transcripts decreased during cold storage, but increased and showed a higher mRNA concentration in ethylene-treated fruit during +2 d on the shelf. Expression of three genes, *PLY1*, *EGase,* and *Exp3* was induced in ethylene-treated fruit at the early stage of cold storage (Figure 5). Since these genes are associated with fruit softening in peach, it is possible that ethylene might promote softening of fruit tissue through inducing the expression of these genes.

### 3.6. Expression of Genes Related to IB

**Figure 6:**
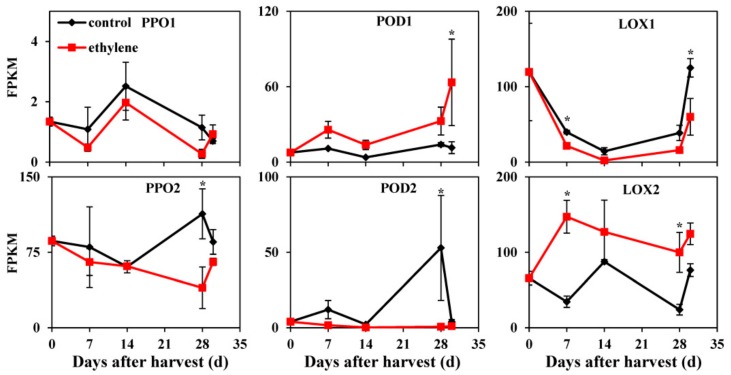
Expression profiles of genes related to internal browning in peach fruit. Data are presented as mean ±SE from three independent biological replicates. Fruit were transferred to 20 °C for 2 d after 28 d of cold storage (28 + 2). Asterisks (*) indicate that mean values are significantly different between ethylene treatment and control (*p* < 0.05) according to Duncan’s multiple range test. LOX, lipoxygenase; POD, peroxidase; PPO, polyphenol oxidase.

*PPO*, *peroxidase* (*POD*), and *lipoxygenase* (*LOX*) gene family members showed different expression levels and/or patterns as well as different responses to ethylene. The IDs of these genes are listed in Table S5. *PPO1* had no significant difference in transcript abundance between control fruit and ethylene-treated fruit, but *PPO2* showed lower transcript abundance in ethylene-treated fruit at 28 d of cold storage (Figure 6). Transcript abundance of *POD1* was higher in ethylene-treated fruit at +2 d on the shelf. On the contrary, *POD2* exhibited an obvious expression peak at 28 d in control fruit but was expressed at quite a low level in ethylene-treated fruit (Figure 6). Two *LOX* members also showed different expression patterns, with higher transcript levels of *LOX1* detected in control fruit at 7 d of cold storage and after transfer to 20 °C, while higher transcript level of *LOX2* were observed in ethylene-treated fruit at 7 and 28 d of cold storage (Figure 6).

### 3.7. Changes in Lipid Diversity and Metabolic Gene Expression 

**Figure 7:**
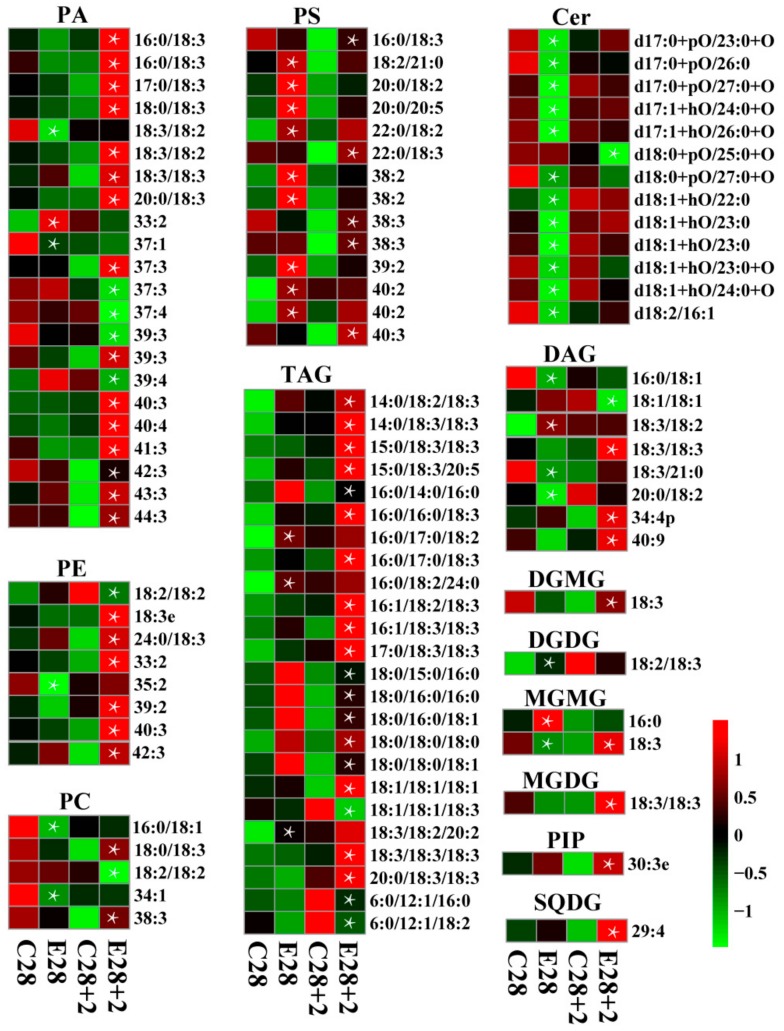
Heatmap of contents of lipids differentially accumulation in the comparison between ethylene and control. The green, black, and red denote the low, middle, and high content. Cer, ceramide; DAG, diacylglycerol; DGDG, digalactosyldiacylglycerol; DGMG, digalactosylmonoacyglycerol; MGDG, monogalactosyldiacylglycerol; MGMG, monogalactosylmonoacylglycerol; PA, phosphatidic acid; PC, phosphatidylcholine; PE, phosphatidylethanolamine; PIP, phosphatidylinositol; PS, phosphatidylserine; SQDG, sulfoquinovosyldiacyglycerol; TAG, triacylglycerol. Asterisks (*) indicate that mean values are significantly different between ethylene treatment and control (*p* < 0.05) according to Duncan’s multiple range test. The sampling points beginning with the letter C or E indicate those belong to control and ethylene treatment, respectively.

**Figure 8:**
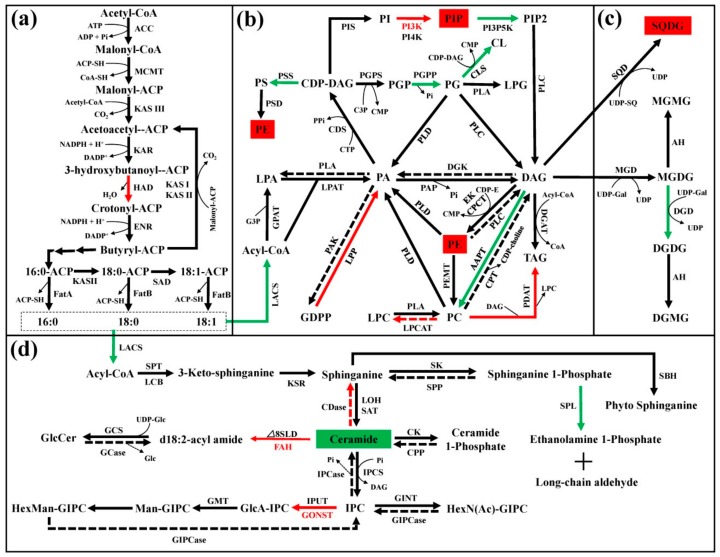
Influence of ethylene on amount of compounds and expression of genes in lipid pathway in peach. The pathway includes four parts as fatty acid biosynthesis (**a**), phospholipid metabolism (**b**), galactolipid metabolism (**c**), and sphingolipid metabolism (**d**). The red arrows indicate promotive effect of ethylene on transcript levels; the green arrows indicate the inhibitive effect of ethylene on transcript levels. Solid arrows indicate biosynthetic steps and dashed ones indicate catabolic steps. The red box indicates lipid with content significantly higher in ethylene treated fruit; the green box indicates lipid with content significantly lower in ethylene treated fruit (*p* < 0.05) according to Duncan’s multiple range test. Δ8SLD, delta8 sphingolipid long-chain base desaturase; AAPT, aminoalcohol phosphotransferases; ACC, acetyl CoA carboxylase; ACP-SH, acyl carrier protein- sulfhydryl; ADP, adenosine diphosphate; AH, acetylhydrolase; ATP, adenosine triphosphate; CDase, ceramidase; CDP-DAG, cytidine diphosphate (CDP)-diacylglycerol (DAG); CDP-E, CDP- ethanolamine; CDS, CDP-diacylglycerol synthase; Cer, ceramide; CK, ceramide kinase; CL, cardiolipin; CLS, CL synthase; CMP, cytidyl monophosphate; CoA, coenzyme A; CoA-SH, coenzyme A-sulfhydryl; CPCT, CTP (cytidine 5′-triphosphate): phosphoethanolamine cytidylyltransferase; CPP, ceramide 1-phosphate phosphatase; CPT, diacylglycerol cholinephosphotransferase; DAG, diacylglycerol; DGAT, DAG acyltransferase; DGD, digalactosyldiacylglycerol synthase; DGDG, digalactosyldiacylglycerol; DGK, DAG kinase; DGMG, digalactosylmonoacyglycerol; DGPP, diacyglycerol pyrophosphate; EK, ethanolamine kinase; ENR, enoyl-ACP reductase; FAD, fatty acid desaturase; FAH, fatty acid alpha-hydroxylase; FatA, Fatty acid thioesterase A; FatB, Fatty acid thioesterase B; G3P, glycerol-3-phosphate; Gal, galactose; GCase, glucosylceramidase; GCS, glucosylceramide synthase; GINT, glucosamine inositolphosphorylceramide transferase; GIPC, glycosyl inositol phosphoceramide; GIPCase, glycosylinositolphosphoceramidase; Glc, glucose; GlcCer, glycosyl ceramide; GMT, GIPC mannosyl-transferase; GONST, golgi-localized nucleotide sugar transporter; GPAT, glycerol-3-phosphate acyltransferase; HAD, hydroxyacyl-ACP dehydrase; HexNAc, nacetylhexosamine; IPC, inositolphosphorylceramide; IPCS, IPC synthase; IPUT, inositol phosphoryl ceramide glucuronosyltransferase; KAR, ketoacyl-ACP reductase; KASI, ketoacyl-ACP synthase I; KASII, ketoacyl-ACP synthase II; KASIII, ketoacyl-ACP synthase III; KSR, ketosphinganine reductase; LACS, long-chain acyl-CoA synthetase; LCB, sphingolipid Δ8 long-chain base; LOH, lag1 longevity assurance homolog; LPA, lysophosphatidic acid; LPAT, lysophosphatidic acid acyltransferase; LPC, lysophosphatidylcholine; LPCAT, LPC acyltransferase; LPP, lipid phosphate phosphatase; Man, mannose; MCMT, malonyl-CoA: ACP malonyltransferase; MGD: monogalactosyldiacylglycerol synthase; MGDG, monogalactosyldiacylglycerol; MGMG, monogalactosylmonoacylglycerol; NADPH, nicotinamide adenine dinucleotide phosphate; PA, phosphatidic acid; PAK, PA kinase; PAP, PA phosphatase; PC, phosphatidylcholine; PDAT, PC: DAG acyltransferase; PE, phosphatidylethanolamine; PEMT, PE methyltransferase; PG, phosphatidylglycerol; PGP, PG phosphate; PGPP, PGP phosphatase; PGPS, PGP synthase; Pi, inorganic phosphate; PI, phosphatidylinositol; PI3K, PI 3-kinase; PI3P5K, PI-3-phosphate-5-kinase; PI4K, PI 4-kinase; PIP, PI phosphate; PIP2, PI bisphosphate; PIS, phosphoinositide synthase; PLA, phospholipase A; PLC, phospholipase C; PLD, phospholipase D; PPi, inorganic diphosphate; PS, phosphatidylserine; PSD, PS decarboxylase; PSS, base-exchange-type phosphatidylserine synthase; SAD, stearoyl-ACP desaturase; SAT, sphingosine N-acyltransferase; SBH, sphingosine base hydroxylase; SK, sphingosine kinase; SPL, sphingosine 1-phosphate lyase; SPP, sphingosine phosphate phosphatase; SPT, serine palmitoyltransferase; SQ, sulfoquinovose; SQD, UDP-sulfoquinovose synthase; SQDG, sulfoquinovosyldiacyglycerol; UDP, uridine 5′-diphosphate; TAG, triacyl glycerol.

Lipids in cell membranes are critical for plants to maintain membrane fluidity, and to acclimatize to cold stress. Therefore, the changes in fatty acid and lipid content during cold storage and +2 on the shelf were analyzed (Figures S4 and S5, Figure 7). It was observed that the content of palmitic acid (16:0), stearic acid (18:0), and oleic acid (18:1) decreased at the beginning of storage and generally remained constant, while linoleic acid (18:2) and linolenic acid (18:3) showed increased trends during cold storage and +2 d on the shelf (Figure S4). However, the contents of these fatty acids showed no significant difference between control and ethylene-treated fruit (Figure S4). Among genes associated with fatty acid biosynthesis, only *hydroxyacyl-ACP dehydrase* (*HAD*) showed higher transcript level in ethylene-treated fruit, other genes including *acetyl CoA carboxylase* (*ACC*), *malonyl-CoA: acyl carrier protein malonyltransferase* (*MCMT*), *ketoacyl-ACP synthase III* (*KASIII*), *ketoacyl-ACP reductase* (*KAR*), *enoyl-ACP-reductase* (*ENR*), and *stearoyl-ACP desaturase* (*SAD*) were not responsive to ethylene treatment (Figure 8a, Figure S6, Table S6). 

A liquid chromatograph mass spectrometer (LC-MS) approach was used for lipid profiling. A total of 24 lipid classes, including 452 lipid species, were detected and quantification data revealed significant ethylene-induced changes in the total content of some lipid classes (Figure S5, Figure 7). While the overall content of 18 lipid classes was not affected by ethylene treatment, an enhanced fatty acid (FA) and a reduced Cer content at 28 d as well as an increased phosphatidylcholine (PE), phosphatidylinositol (PIP), ceramide phosphatidylinositols (CerP), and sulfoquinovosyldiacyglycerols (SQDG) content at 28 + 2 d was observed in ethylene-treated fruit (Figure S5). 

The accumulation of some phospholipids was affected by ethylene treatment. Among 22 differentially accumulated phosphatidic acids (PAs), 16 PA species showed higher levels in ethylene-treated fruit (Figure 7). The content of six, out of eight, PEs was also higher in ethylene-treated fruit on shelf (Figure 7). Meanwhile, ethylene-induced increment in the content was found for PIP and all 14 phosphatidylserine (PS) species at 28 d of cold storage and/or at 2 d on shelf (Figure 7). Higher levels of most triacylglycerols (TAGs) were observed in ethylene-treated fruit. Accumulation of some phosphatidylcholines (PCs) and some diacylglycerols (DAGs) was promoted by ethylene treatment while that of some others was inhibited. For the glyceroglycolipids, a significant increase in content of digalactosyldiacylglycerol (DGDG) and a monogalactosyldiacylglycerol (MGMG 16:0) at 28 d of cold storage, digalactosylmonoacyglycerol (DGMG), another MGMG (18:3), monogalactosyldiacylglycerol (MGDG), and SQDG at +2 d on the shelf were observed (Figure 7). 

The expression of all genes involved in phospholipid and glyceroglycolipid metabolism is shown in Figure 8, Figure S6, Table S6. Significant changes in expression of some genes were observed. Fatty acid desaturases (FAD), although not illustrated in Figure 8, are involved in lipid metabolic pathways by catalyzing desaturation process of various lipids, including phospholipids, glyceroglycolipids, and sphingolipids. Expression of five *FAD* gene members was detected in this study and the expression of *FAD3* (Prupe7G261900) was induced following ethylene treatment (Figure S7). Free fatty acids are converted to acyl-CoAs in reactions catalyzed by long-chain acyl-CoA synthetase (LACS). In this study, ethylene increased the expression level of *LACS1* at 14 d of cold storage but inhibited the expression level of *LACS2* during cold storage and +2 d on the shelf (Table S6, Figure S6). Generally, *LACS* showed lower expression level in ethylene-treated fruit (Figure 8, Table S6). Another gene with generally lower transcript abundance in ethylene-treated fruit is *aminoalcohol phosphotransferase* (*AAPT*), encoding the enzyme catalyzing the synthesis of PC from DAG. In plants, phosphatidic acid phosphatase (PAP) catalyzes PA to form DAG. Expression of *PAP* members was distinctly affected by ethylene treatment, with *PAP2* (Prupe.7G158200) significantly down-regulated while *PAP4* (Prupe.3G148800) significantly up-regulated (Figure S6, Table S6). Lower transcript levels of *AAPT*, *PAP2 and phosphatidylinositol-3-phosphate-5-kinase* (*PI3P5K*), as well as higher transcript level of *lipid phosphate phosphatase 1* (*LPP1*), *FAD*3, *PC: DAG acyltransferase 2*, *lysophosphatidylcholine acyltransferase* (*PDAT*), *lysophosphatidylcholine acyltransferase* (*LPCAT*), *PI 3-kinase* (*PI3K*), and *PAP4* were observed in ethylene-treated fruit (Figure 8b, Figure S6, Table S6) and matched well with the change in content of some individual phospholipid species (Figure 7). 

Similar to phospholipids, alterations of sphingolipids metabolism were also induced by ethylene. When analyzing sphingolipids classes, a marked reduction in all Cer was observed in ethylene-treated fruit (Figure 7). 

### 3.8. Identification of Coexpressed Gene Modules 

**Figure 9:**
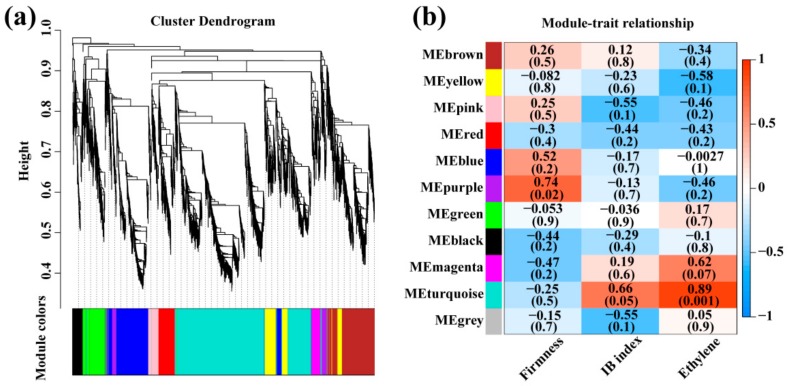
Network analysis dendrogram showing modules identified by weighted gene co-expression network analysis (WGCNA). (**a**) Dendrogram with color annotation; (**b**) correlation analysis between the module eigengene and physiological traits. Each cell contains the corresponding correlation and *p*-value. The left panel shows nine modules. The color scale on the right shows module-trait correlation from −1 (blue) to 1 (red).

To obtain a comprehensive understanding of gene expression differences between control and ethylene–treated fruit, and to identify the specific genes highly associated with CI, a WGCNA was performed. Genes with twofold difference in expression between ethylene treatment and control for at least one time point (*p* < 0.05), 4350 in total, were used for the WGCNA. Eleven co-regulated gene modules, designated by colors, were identified (Figure 9a). The number of genes per module ranged from 1 (MEgrey) to 1683 (MEturquoise). To identify important genes associated with CI, module-trait associations were quantified using the eigengene profile for each module. MEpurple and MEblue were positively correlated to firmness, with a coefficient of 0.74 and 0.52, respectively (Figure 9b). MEturquoise exhibited a high positive correlation, whereas MEpink exhibited a negative correlation to IB index, with coefficients of 0.66 and −0.55, respectively. The positive correlations between ethylene and MEturquoise exhibited the highest coefficient, 0.89, and a lowest *p*-value, 0.001 among all module-trait correlations. The highest number of ERFs was found in MEturquoise among all modules and is described in detail below.

### 3.9. Changes in Transcript Abundance of Ethylene Response Factors (ERFs) 

**Figure 10:**
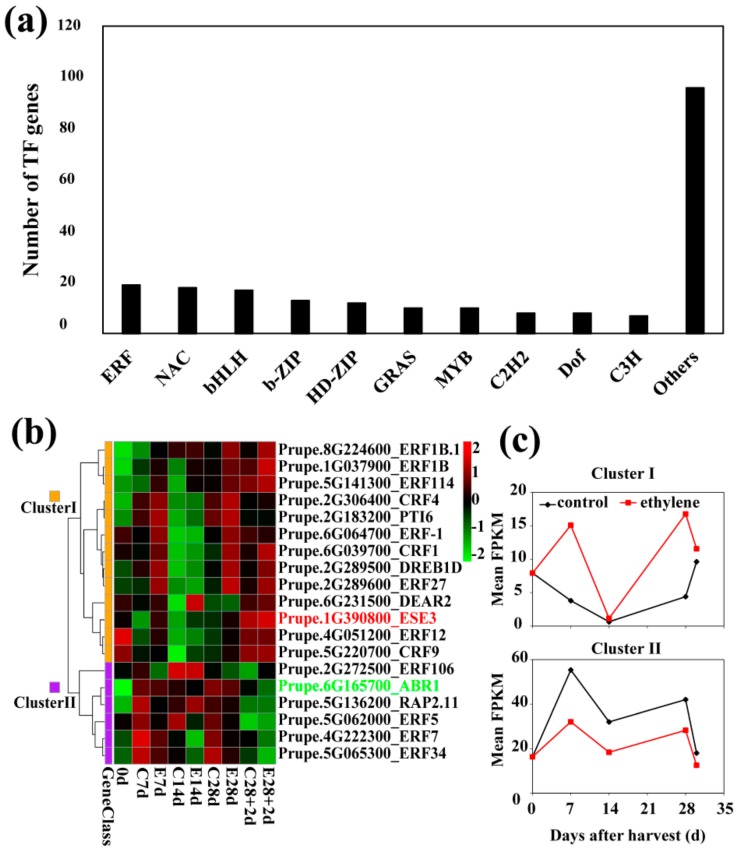
Transcription factors differentially expressed in control and ethylene fruit. (**a**) The number of differentially expressed transcription factors from different classes. (**b**) Expression profiles (log_2_ FPKM) of differentially expressed *ERF* family members. Red letter indicates gene transcript level was significantly up-regulated on shelf; green letter indicate gene transcript level was significantly induced by low temperature storage. (**c**) Representative expression patterns of mean FPKM value of genes in two clusters from hierarchy cluster analysis in (**b**). ABR, abscisic acid repressor; CRF, cytokinin response factor; DEAR, DERB and EAR motif protein; DREB, dehydration responsive element binding factor; ERF, ethylene response factor; FPKM, fragments per kilobase of exon per million reads mapped; PTI, pathogenesis-related genes transcriptional activator. The sampling points beginning with the letter C or E indicate those belong to control and ethylene treatments, respectively. The two ERF members with IDs and names in red or green are those further described in detail in the following analyses.

Among all 4350 DEGs (Figure 2a), a total of 40 transcription factor (TF) families, including 218 differentially expressed TFs, were identified. The top 10 family members accounted for over half of all differentially expressed TFs (Figure 10a). The *ERFs* were the family with the highest number (19) of differentially expressed members. HCA was used to analyses the change in *ERFs* in ethylene-treated fruit during cold storage and these 19 *ERFs* were sorted into two clusters based on HCA analysis (Figure 10b). Members in Cluster I showed different expression profiles between ethylene-treated and control fruit, but transcript levels were consistently higher in ethylene-treated fruit (Figure 10c). In contrast, *ERFs* in Cluster II showed similar expression trends between ethylene-treated and control fruit, with lower transcript levels of *ERFs* in ethylene-treated fruit (Figure 10c).

### 3.10. Candidate ERFs Involved in Regulating Cell Wall and Lipid Metabolism

As described above and shown in Figure 9b, MEturquoise is the module having the highest coefficient with ethylene production as well as with IB index. This module contains 1683 genes, including cell wall related genes, lipids gene, and highest numbers of *ERFs*, suggesting a potential correlation between *ERFs* and genes involved in cell wall and lipid metabolism.

**Figure 11:**
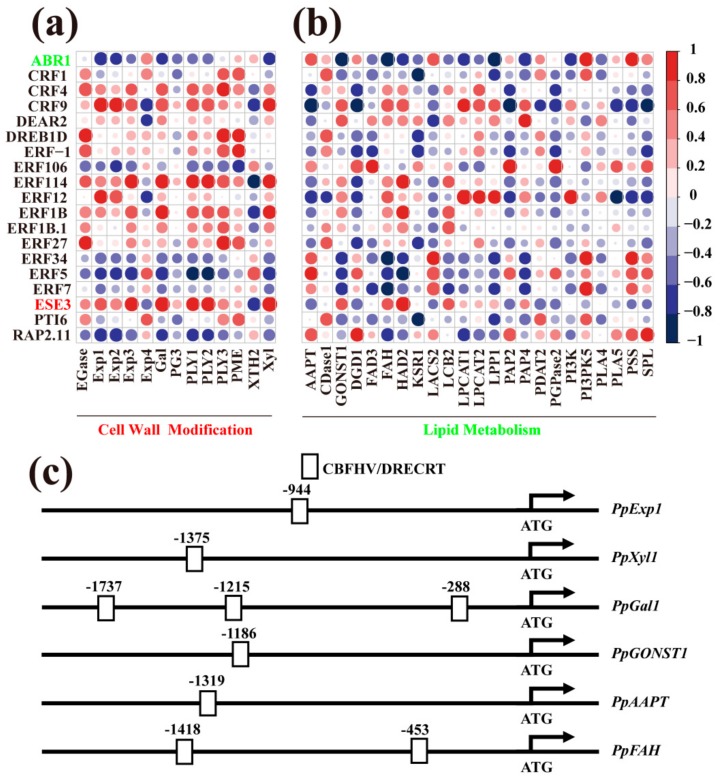
Correlation between differentially expressed *ethylene response factors* (*ERFs*) and differentially expressed cell wall related genes (**a**), lipid metabolism genes (**b**). Schematic diagram of the *Exp1*, *Xyl*, *Gal*, *GONST1*, *AAPT,* and *FAH* promoter showing the potential ERF-binding sites. Prediction of the *cis*-acting elements in the 2000-bp *Exp1*, *Xyl*, *Gal*, *GONST1*, *AAPT,* and *FAH* promoter region was performed by searching the PLACE databases (**c**). *ESE3* and *ABR1*, with expression highly correlated with cell wall modification and lipid metabolism related genes, respectively, were indicated in red and green, respectively. The color gradient from blue to red indicates increase of correlation from –1 to 1. The abbreviations are defined in the legends to Figure 5, Figure 8 and Figure 10.

In MEturquoise, ten cell wall related genes (*PG3*, *Exp1*, *Exp2*, *Exp3*, *Exp4*, *PLY1*, *PLY2*, *Xyl*, *Gal,* and *XTH2*) were identified as being coexpressed with *ERFs* (*ESE3*, *CRF4*, *ERF1B*, *CFR9*, *ERF1B.1*, *ERF114,* and *ERF5*; Figure S8). In MEturquoise, the transcript level of *ESE3* was highest among all *ERFs* in fruit at +2 d on the shelf (Table S7) and also showed a high weight value (Figure S8) and correlation (Figure 11a) with the expression levels of cell wall related genes. The transcript level of *ESE3* showed a 146-fold increase after transfer to the shelf for +2 d (Figure 10b, Table S7). A phylogenetic tree comparison using *Arabidopsis ERF* family members and peach differentially expressed *ERFs* showed a close relationship between peach ESE3 and the *Arabidopsis* counterpart (Figure S9). A *cis*-element analysis revealed several CBFHV/DRECRT response elements in the promoters of cell wall metabolism related genes, *Exp1*, *Xyl,* and *Gal* (Figure 11c). Other cell wall related genes, including *PLY3*, *PME,* and *EGase* were coexpressed with *CRF4*, *PTI6*, *ERF-1*, *ERF27,* and *DREB1D*, respectively in the green module, but showed a lower correlation with ethylene production (Figure 9b, Figure S8). Moreover, CBFHV/DRECRT response elements were not found in the promoter of *PLY3*, *PME,* and *EGase*. These data indicate that ESE3 is the potential ERF member binding to the promoter of *Exp1*, *Xyl,* and *Gal* and contributes to ethylene-promoted fruit softening processes. 

Compared with other *ERFs*, the transcript level of *ABR1* was significantly induced by low temperature and showed a 105-fold increase during the first week in cold storage (Figure 10b, Table S7). Correlation analysis showed that expression of *ABR1* is highly positively correlated with that of *AAPT* and highly negatively correlated with those of *GONST1*, *FAH*, and *LPP1* (Figure 11b). Phylogenetic tree comparisons showed a close relationship between peach ABR1 and the *Arabidopsis* counterpart (Figure S9). ERF binding sites were identified in promoters of *AAPT, FAH, GONST1,* and *AAPT* (Figure 11c). Other *ERFs* were also found to co-express with lipid metabolism related genes in the MEturquoise and other modules (Figure S8). These results suggested that lipid genes *AAPT, FAH, GONST1,* and *AAPT* might be regulated by ABR1 and all these genes are involved in ethylene-alleviated fruit CI.

## 4. Discussion

### 4.1. Ethylene is An Important Regulator of Postharvest Chilling Injury in Fruits

**Figure 12:**
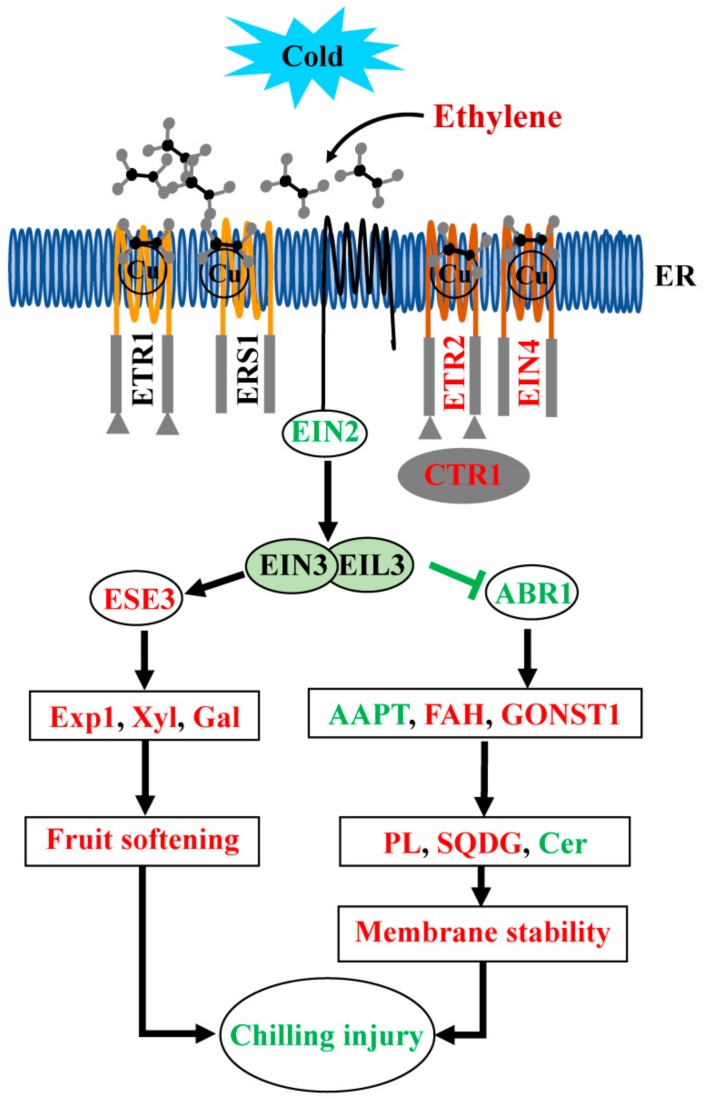
Model of the effect of ethylene on the alleviation of chilling injury via ERF signaling pathway. The genes, enzymes and processes in red denote promotive effects of ethylene, with those in green refer to inhibitive effects, as indicated by difference analysis for gene expression and metabolites using one-way ANOVA. ER, endoplasmic reticulum; PL, phospholipid. Other abbreviations are defined in the legends to Figure 4, Figure 5, Figure 8 and Figure 10.

Ethylene plays important roles in response to biotic and abiotic stimuli and stresses in plants [50]. Effect of ethylene on CI is still controversial although a series of reports regarding it have been published. On the one hand, ethylene alleviates CI in banana and citrus fruits. Exogenous ethylene significantly induced the chilling tolerance in harvested banana fruit [51], and CI was reduced by applying 2 μL L^−1^ ethylene in citrus fruit during storage at 1.5 °C [52]. On the other hand, opposite effects of ethylene on CI have also been reported. For instance, treating Charentais cantaloupe melons with ethylene (10 μL L^−1^) before cold storage aggravated the development of CI [53]. The effect of ethylene on the incidence of CI is even dubious within one species. In peach, for example, involvement of endogenous ethylene production in the alleviation of postharvest fruit CI was reported in previous studies [1], and applying exogenous ethylene treatment markedly alleviated fruit CI [26,27]. However, the alleviation of CI by blocking ethylene action with 1-MCP was also found [29,30]. Such difference in ethylene effects on CI might be due to differences in species or variety, as well as ethylene treatment manners and application time in the different studies. The current study found that ethylene treatment alleviated peach fruit CI symptom (Figure 1). Based on the data obtained, a model is proposed to clarify the underlying mechanism of ethylene in alleviating CI (Figure 12). The ethylene signal is activated by high ethylene concentrations, which then induces the expression of some *ERFs*. The activated ERFs might regulate fruit softening and alterations in lipid components by controlling the expression of genes in the cell wall and lipid metabolism pathway.

### 4.2. Alleviation of Postharvest Chilling Injury by Ethylene Involves Altered Expression of Genes Related to Browning and Cell Wall Metabolism

PPO and POD are major enzymes responsible for the browning of plant tissues by oxidizing phenolic substrates, and LOX is a candidate participant in membrane alteration and lipid degradation [54]. Increased PPO and LOX activities were observed at chilling temperatures in banana fruit and maize seedlings [55,56]. Aggravated CI symptoms following ethylene treatment in conjunction with low temperature in cantaloupe melons was associated with a reduction in POD activity [53]. In this study transcript abundance of *PPO1*, *PPO2,* and *POD2* was reduced when the fruits were exposed to ethylene (Figure 6). Previously we observed that lower transcript levels of *PPOs* and higher production of ethylene were also involved in alleviating CI symptoms by LTC [1]. POD activity of Fortune mandarins fruit held under an ethylene atmosphere with a less CI was significantly lower than that of fruit stored in air at low temperature [52]. Therefore, a lower transcript level of *PPOs* and *POD2* in ethylene-treated fruit could be defensive responses to alleviate CI symptoms. 

Our data also showed different expression patterns of *LOXs* in peach fruit exposed to ethylene. Higher expression level of *LOX2*, the ortholog of *TomloxB,* was observed in ethylene-treated fruit during cold storage and lower expression of *LOX1*, the ortholog of *TomloxA,* was found in ethylene-treated fruit at +2 d on the shelf (Figure 6). This is similar to what has been reported previously in tomato, where expression of three *LOX* members (*TomloxA*, *TomloxB,* and *TomloxC*) responded differently to ethylene treatment during fruit ripening, which indicates a dual role of LOX in fruit development including a defensive component and a contribution to aroma and flavor generation [57]. Therefore, the increased expression of *LOX2* in ethylene-treated peach fruit could maintain the integrity of plasma membrane lipids and contribute to the defence against cold stress, while decreased expression of *LOX1* in ethylene-treated fruit might be related to other biological processes, possibly loss of aroma, which is another symptom of chilling-injured peach fruit [58].

Aside from browning, failure to soften is also a major CI symptom in peaches. Abnormal cell wall dismantling is associated with flesh softening at low temperature [1]. Thus, the levels of transcripts encoding proteins involved in cell wall metabolism were analyzed in this study. *PLY1*, *PLY3*, *PME*, *EGase*, *Xyl*, *Exp1*, *Exp3*, *Gal,* and *GS* genes exhibited increased transcript levels in ethylene-treated fruit that matched the reduction in firmness (Figure 5), which is similar to the results of our previous study that restoration of normal softening of LTC-treated fruit is associated with the higher transcript levels of these genes [1]. However, in the present experiments, the transcripts abundance of *PLY2*, *Exp2*, *XTH1,* and *XTH2* mRNA remained unchanged in fruit exposed to ethylene (Figure 5), which indicate that cell wall metabolism genes might be regulated by multiple factors. Moreover, in the present study, the higher levels of cell wall metabolism gene transcripts in ethylene-treated fruit (Figure 5) are consistent with previous studies where the mRNAs of *PG*, *Xyl,* and *Exp3* were up-regulated by ethylene treatment in peach [15] and the activity of PG, PME, and Gal was prevented by 1-MCP in chilling-induced abscission of *Dendrobium* flowers [59]. Overall, this suggests that up-regulation of genes related to cell wall metabolism, causing a moderate loss of firmness in peach fruit (Figure 5), might be controlled by ethylene.

### 4.3. Role of Lipid Rearrangements in Regulating Cold Tolerance

The development of CI symptoms is closely related to dysfunction of cell membranes under cold stress. A higher proportion of unsaturated fatty acids in lipids is beneficial for maintaining membrane fluidity to block the occurrence of CI [8]. Besides, phospholipids and sphingolipids metabolism also change in response to cold stress [60]. However, the mechanism whereby ethylene regulates lipid metabolism during low temperature storage remains unclear. Our results provide information about ethylene-induced metabolic and transcript dynamics regarding different lipid species during cold storage and +2 d shelf life (Figure 7 and Figure 8). A previous study has shown that the maintenance of high levels of membrane lipid desaturation attenuated the CI in fruit [61]. For example, treatment of peach fruit with LTC increased the resistance to CI and was accompanied by an increase in polyunsaturated fatty acids (18:2 and 18:3) contents and a decrease in monounsaturated fatty acid (18:1) content [1]. Unlike previous studies [1,8], the results presented here showed that the content of saturated and unsaturated fatty acids did not change significantly between ethylene and control, which is consistent with gene expression data, i.e., no significant change in transcript levels of genes involved in the fatty acid biosynthetic pathway (Figure 8a, Figure S6). These results suggested that the effect of ethylene on chilling resistance is not likely by regulating fatty acid metabolism. Therefore, different treatments achieve the alleviation of CI via different mechanisms.

The changes in phospholipid content occurring during cold acclimation are important for maintaining membrane integrity [62]. Higher levels of PAs, PEs, PIPs, PSs and a higher saturation of PCs (38:3, 18:0/18:3), as well as a lower saturation of PCs (16:0/18:1; 18:2/18:2; 34:1) were observed in ethylene-treated fruit compared with those in control fruit (Figure 7). In *Arabidopsis*, the formation of PA was found to occur via the PLC-DGK (phospholipase C-DAG kinase) and PLD (phospholipase D) pathways under cold stress [63]. It is also produced via the action of LPP, which phosphates diacyglycerol pyrophosphate (DGPP) to generate PA [64]. In this study, elevated levels of PAs contribute to the alleviation of IB by ethylene (Figure 7), which is consistent with a higher PA level in LTC-treated fruit during cold storage [1]. Since the *PLD*, *PLC,* and *DGK* transcripts did not change, a higher PA accumulation might be associated with a higher transcript level of *LPP* (Figure 8, Figure S6). Previously, enhanced PA has been reported to decrease damage caused by reactive oxygen species under freezing stress in *Arabidopsis* [65]. Phospholipids also play positive roles in cold tolerance, for example, the relative amounts of PE increased in *Arabidopsis* after two weeks under cold stress [62]. In the present study, the total PE increased in ethylene-treated fruit after transfer to 20 °C (+2 d) (Figure S5). This could alleviate the damage to cell membrane and contribute to IB alleviation. Meanwhile, ethylene-treated peach showed higher level of PS (Figure 7), and PS biosynthesis is required for cold stress response in *Arabidopsis* [66]. For the PCs, down-regulation of *AAPT* and up-regulation of *FAD3* transcript levels might be directly correlated with the decreased content of a lower unsaturation of PCs and the increased content of a higher unsaturation of PCs, respectively (Figure 7 and Figure 8, Figures S5 and S6). Meanwhile, PC could also be regarded as a biomarker in cold stress [67], it can be suggested that phospholipid metabolic changes probably contributed to alleviation of CI by ethylene under low temperature stress in peach fruit (Figure 12).

The levels of TAGs are functionally connected to plant cold tolerance [62,68]. TAG can be produced from DAG by DAG acyltransferase (DGAT) under freezing conditions [69]. The total concentration of TAG and DAG did not change between ethylene-treated fruit and control (Figure S5), but dramatic increases in the composition of TAG and DAG likely reflected an enhanced cold tolerance (Figure 7). In *Cuphea*, crystallized TAGs may lead to damage of cellular structures and cell death in seeds during low-temperature storage [68], and in *Arabidopsis*, exogenous application of DAG could enhance the freezing tolerance of *sag101*, *eds1,* and *pad4* mutants [70]. Therefore, increasing TAG and DAG contents could be an important strategy for peach to adapt to cold stress.

In general, the amount of Cers localized in the plasma membrane correlated negatively with freezing tolerance of leaves after acclimation [62]. In addition to the observed change in the phospholipid levels after ethylene treatment, a change in the relative content of sphingolipids was also observed in peach and a decrease in the content of Cer was associated with the alleviation of CI by ethylene (Figure 7). Cer biosynthesis is catalyzed by ceramide synthase (LOH), but this reaction can be reversed by CDase. Meanwhile, Cer can also be hydroxylated to d18:2-acyl amide by FAH and △8SLD, and converted to glycosyl inositol phosphoceramides (GIPCs) by a series of enzymes including GONST. Thus, the higher transcript levels of *FAH, CDase1,* and *GONST1* revealed by the transcriptome analysis might be expected to result in decreased Cer biosynthesis in ethylene-treated fruit (Figure 8d). In plants, high level of Cer decreases cold tolerance by reducing membrane fluidity and leads to the death of plant cells [71,72]. Thus, a low level of Cer in ethylene-treated fruit may alleviate the damage to cell at low temperature.

Galactolipids are important for maintaining the stability of membranes in *Arabidopsis* and lichen during cold stress conditions [10,60]. The degradation of galactolipids is associated with selective breakdown of plastid membranes in apple flesh [73]. MGDG levels increased in *Arabidopsis* under chilling [74]. In peach, increased galactolipid levels were found in resistant varieties, which significantly correlated with the tolerance to CI [75]. Additionally, an increased MGDG and DGDG contents are responsible for the alleviation of CI in guava fruit exposed to 5 kPa CO_2_ [76]. In this study, we observed increased galactolipid (MGDG, DGDG, MGMG, DGMG, and SQDG) levels in ethylene-treated fruit (Figure 7). Thus, it might be considered that galactolipids species play a key role in ethylene-alleviation of the CI process, and lipid remodeling is required for ethylene-alleviated CI, operating by protecting membrane integrity in peach fruit (Figure 12).

### 4.4. Cell Wall and Lipid Metabolism are Regulated by Different ERF Members

The ERFs belong to the AP2/ERF superfamily of TFs and play important roles in various physiological processes, including cold-stress responses [18,19]. In *Arabidopsis*, expression of DREB1/CBF members is induced by low temperature and these members are likely to be major regulators of the response to cold stress [19]. In the present study, a total of 19 differentially expressed *ERFs* with two distinct expression patterns were identified in response to ethylene treatment (Figure 10b,c). Coexpression, correlation and *cis*-element analysis showed that ESE3 was potentially involved in regulating expression of cell wall metabolism genes, *Exp1*, *Xyl,* and *Gal* (Figure 11a, Figure S8). Peach ESE3 has a high sequence identity to ESE3, SHN1, SHN2, and SHN3 from *Arabidopsis* (Figure S9). In *Arabidopsis*, SHNs have been reported to be involved in regulating metabolism of lipid and/or cell wall components during drought stress [77]. Furthermore, expression of the *Arabidopsis* ortholog of *ESE3* is induced by salt, ACC, or ethylene and is reduced by aminoethoxyvinylglycine (AVG), an inhibitor of ethylene biosynthesis or AgNO_3_, an inhibitor of ethylene perception [78]. In our study, the transcripts of *ESE3* increased at least 2-fold at each time point in ethylene-treated fruit (Figure 10b, Table S7), which suggests that ESE3 may be required for ethylene-alleviated CI progress. Coexpression results showed high weight value between *ESE3* and cell wall genes *Exp1*, *Xyl*, and *Gal* (Figure S8, Figure 11a) and CBF/DRE binding sites were found in the promoters of these cell wall genes (Figure 11c). Therefore, ESE3 might be induced by ethylene and bind to the CBF/DRE sites in the *Exp1*, *Xyl*, and *Gal* promoters to induce their expression to regulate fruit softening (Figure 12).

Among all differentially expressed *ERFs*, *ABR1* is the sole member significantly highly correlated with expression of key lipid genes *AAPT*, *LPP1*, *FAH*, *CDase1,* and *GONST1* (Figure 11b). Peach ABR1 has the highest sequence identity to *Arabidopsis* ABR1 (Figure S9), which has been reported to be responsive to ABA and stress conditions including cold, high salt, and drought during seed germination in *Arabidopsis* [79]. ABR1 acted as a novel negative regulator of abscisic acid signaling and is essential for cell death [80]. In this study, *ABR1* showed a 3.2-fold decrease in ethylene-treated fruit transferred to 20 °C for 2 d (Figure 10b, Table S7). Interestingly, *cis*-element analysis showed that several CBF/DRE response elements were present in the promoters of *AAPT*, *FAH,* and *GONST1* (Figure 11c). These results suggest that the ethylene-induced decrease in *ABR1* expression might result in alleviation of CI by regulating lipid metabolism. Furthermore, in our previous study, it was observed that expression of *ABR1* was inhibited by LTC in peach and ABR1 may act as a repressor in regulating the expression of lipid genes [1]. Therefore, these results also support the idea that ethylene is involved in the change of lipid metabolism during LTC-alleviated CI process, and that ABR1 might play a critical role in this process.

## 5. Conclusions

On the basis of our results and knowledge from the literature, we propose a hypothetical working model for alleviation of postharvest peach fruit CI by ethylene. The details of ethylene perception and response are known from other studies. Ethylene is perceived by its receptors, ETR2 and EIN4, which then results in the degradation of EIN2 phosphorylated by CTR1, and consequently, induces the accumulation of ESE3. Increased ESE3 then might bind to the CBF/DRE site of *Exp1*, *Xyl,* and *Gal* promoters to induce expression of these genes to promote fruit softening. At the same time, EIN3/EIL3 inhibits the accumulation of ABR1, and then inhibits the expression of *AAPT*, but enhances the expression of *FAH* and *GONST1* to promote the biosynthetic metabolism of phospholipids (PL) and glycerolipids (especially SQDG) and the catabolic reaction of Cer. Adjustment of lipids maintains the stability of membrane, which may contribute to alleviating the development of CI. Our findings in this study provide new insights into the role and mechanisms of ethylene in regulating postharvest fruit CI and have implications for improving cold storage techniques with regard to ethylene manipulation.

## Supplementary Materials

The following are available online at https://www.mdpi.com/2073-4409/8/12/1612/s1, Figure S1: Principal component analysis (PCA) of fruit at harvest, after cold storage and subsequent ripening on shelf (+2 d) on the basis of transcriptome data, Figure S2. Gene ontology (GO) analysis of differentially expressed genes (DEGS), Figure S3: The correlation analysis between the relative expression data from real time quantitative PCR and FPKM in RNA-Seq results in control and ethylene-treated fruit, Figure S4: The effect of ethylene on fatty acid levels in peach fruit, Figure S5: Total content of lipids in control and ethylene-treated fruit at 28 d of cold storage and +2 d of shelf, Figure S6: Expression profiling of genes related to lipid metabolism under ethylene treatment and in the control, Figure S7: The effect of ethylene on expression of *FADs* in peach fruit, Figure S8: Coexpression network between *ERFs* and genes related to cell wall modification lipid metabolism, ethylene biosynthesis and internal browning, Figure S9: Phylogenetic tree of identified ERFs, Table S1: Primers for real-time quantitative PCR, Table S2: Quality of reads for 27 RNA-Seq samples, Table S3: Mapping statistics for 27 RNA-Seq samples, Table S4: Correlation matrix between 27 RNA-Seq samples, Table S5: List of names and IDs of genes involved in ethylene biosynthesis and signaling, cell wall modification, and browning, Table S6: The results of multiple comparison of lipid gene expression after one-way ANOVA at 5% significance level, Table S7: Expression profiles (FPKM) of differentially expressed *ERFs*.
