# Supplementary material for: Effect of Ethylene on Cell Wall and Lipid Metabolism during Alleviation of Postharvest Chilling Injury in Peach
Source: Cells. 2019 Dec 11;8(12):1612. doi: 10.3390/cells8121612 (PMC6952997; doi:10.3390/cells8121612)
Supplement: Supplementary file 1 [file cells-08-01612-s001.pdf]

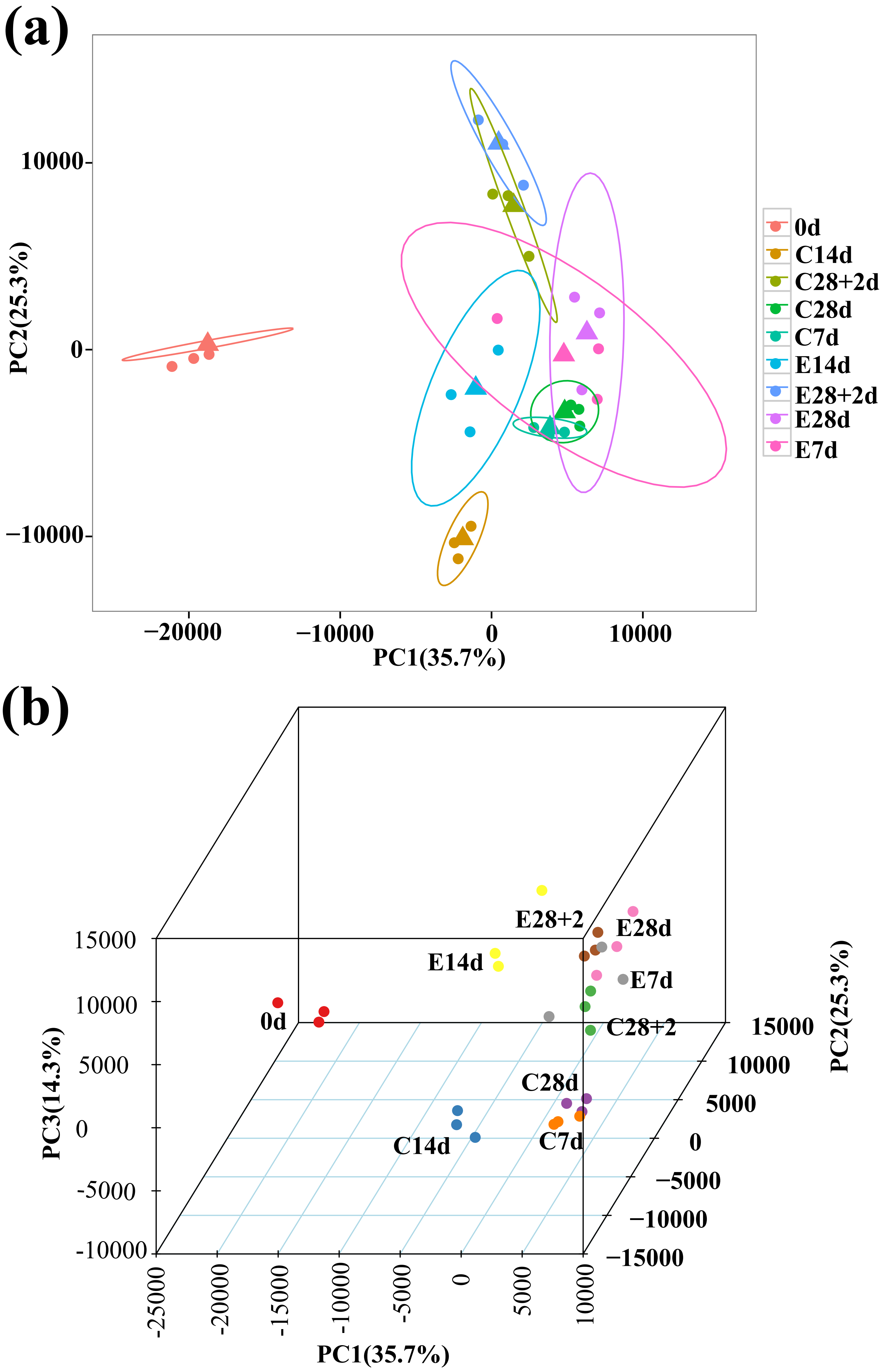

Figure S1. Principal component analysis (PCA) of fruit at harvest, after cold storage and subsequent ripening on shelf (+2 d) on the basis of transcriptome data. (a) PC1 vs PC2; (b) PC1 vs PC2 vs PC3. Three replicates per sample were analyzed. The percentage in the axis was explained by each PC. 0d, harvest point; 28+2 d,subsequent ripening for 2 d after 28 d of cold storage. PC, principal component. The sampling points beginning with letter C or E indicate those belonging to control and ethylene treatments, respectively.

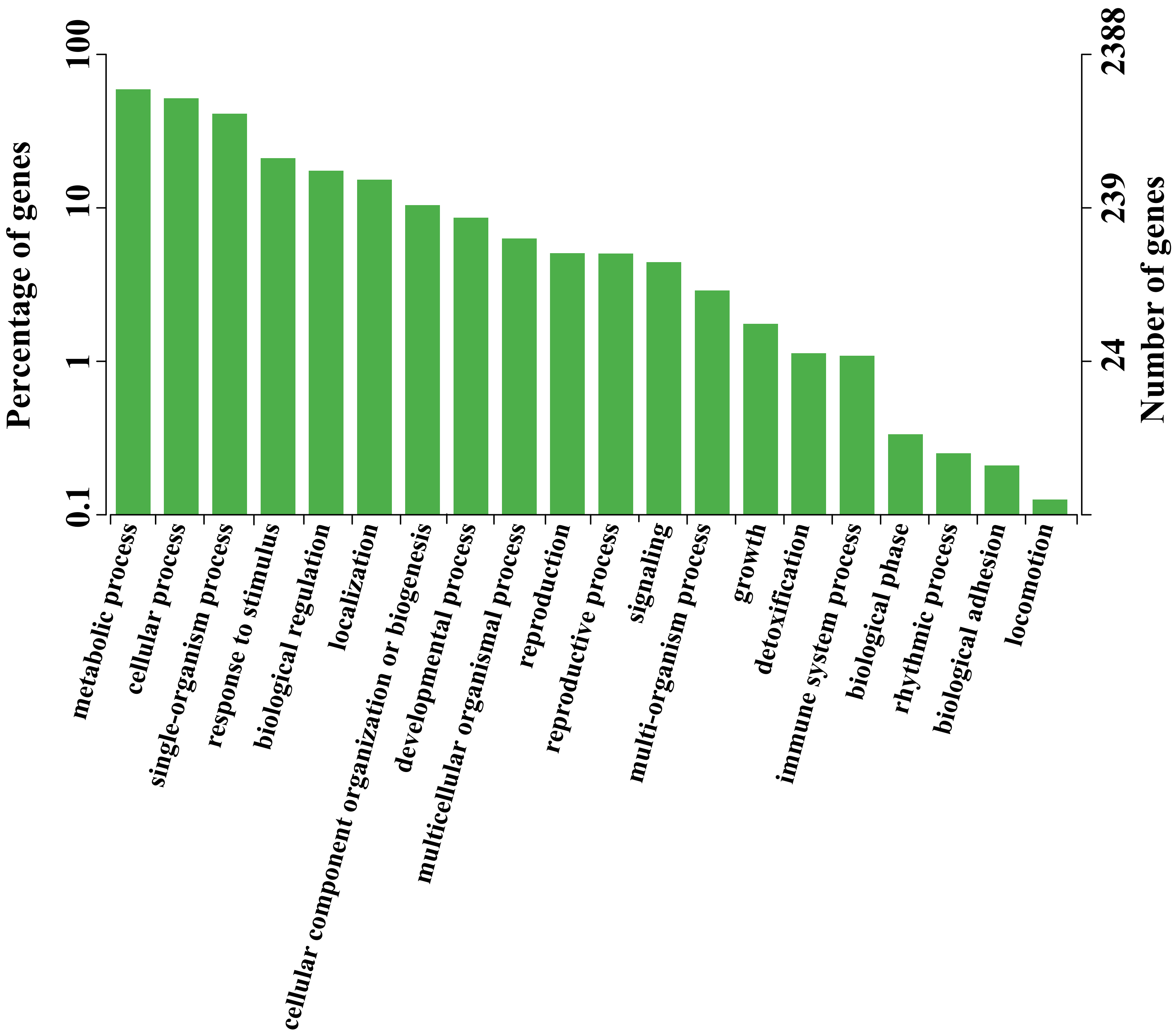

Figure S2. Gene Ontology (GO) analysis of differentially expressed genes (DEGS).

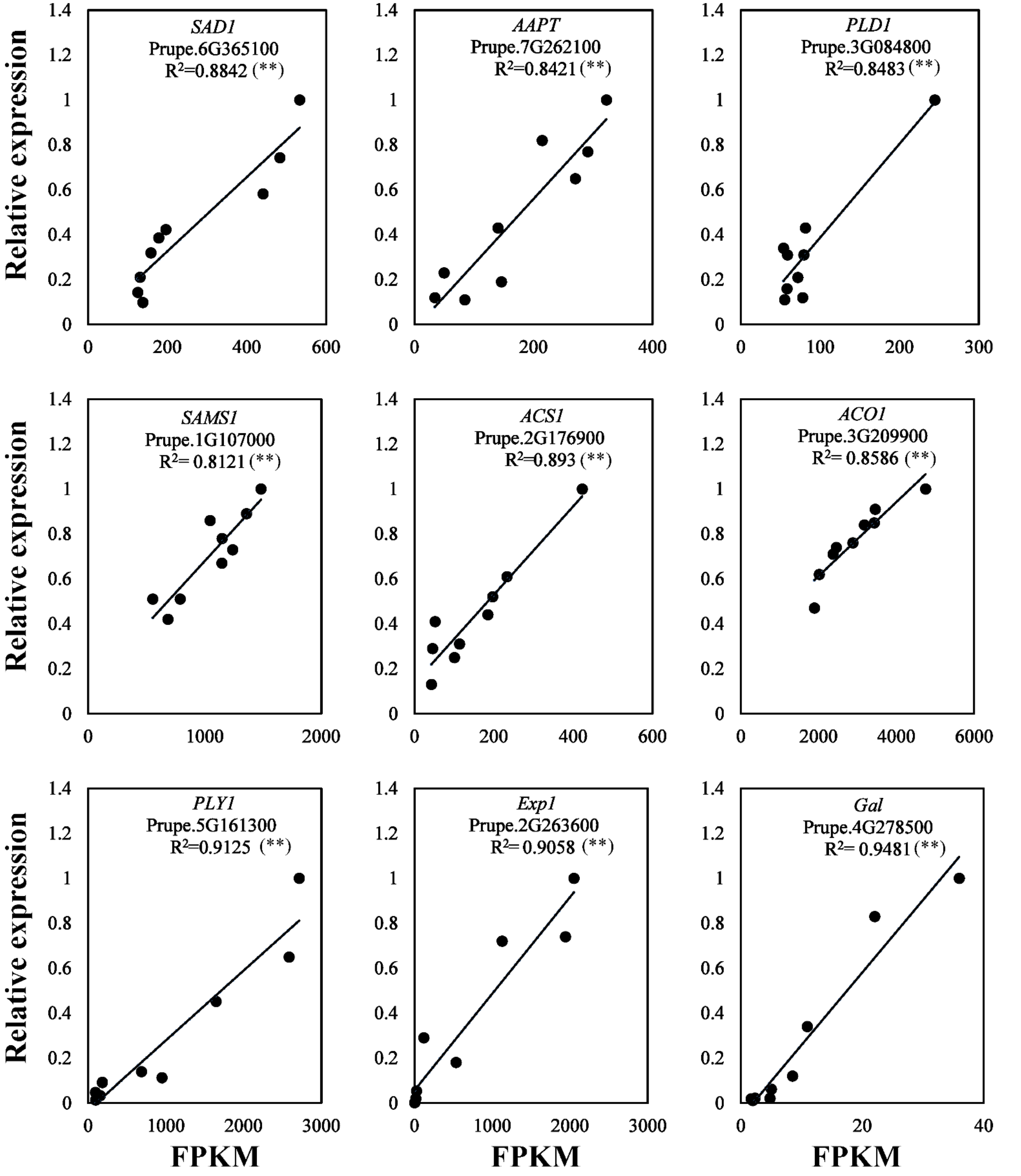

Figure S3. The correlation analysis between the relative expression data from real time quantitative PCR and FPKM in RNA-Seq results in control and ethylene-treated fruit. The genes were chosen representing lipids metabolism, ethylene synthesis metabolism and cell wall metabolism. Line represents the orthogonal fit to the data and correlation (R) is shown. FPKM, reads per kilobase per million mapped reads. Abbreviations used are as follows: SAD, stearoyl-ACP desaturase; AAPT, aminoalcoholphosphotransferases; PLD, phospholipase D; SAMS, S-adenosylmethionine synthase; ACS, ACC synthase; ACO, 1-aminocyclopropane-1-carboxylic acid (ACC) oxidase; PLY, pectate lyase; Exp, expansin; Gal, galactosidase. Asterisk indicates a significantly correlation between relative expression level and FPKM ($P < 0.01$) according to F-test.

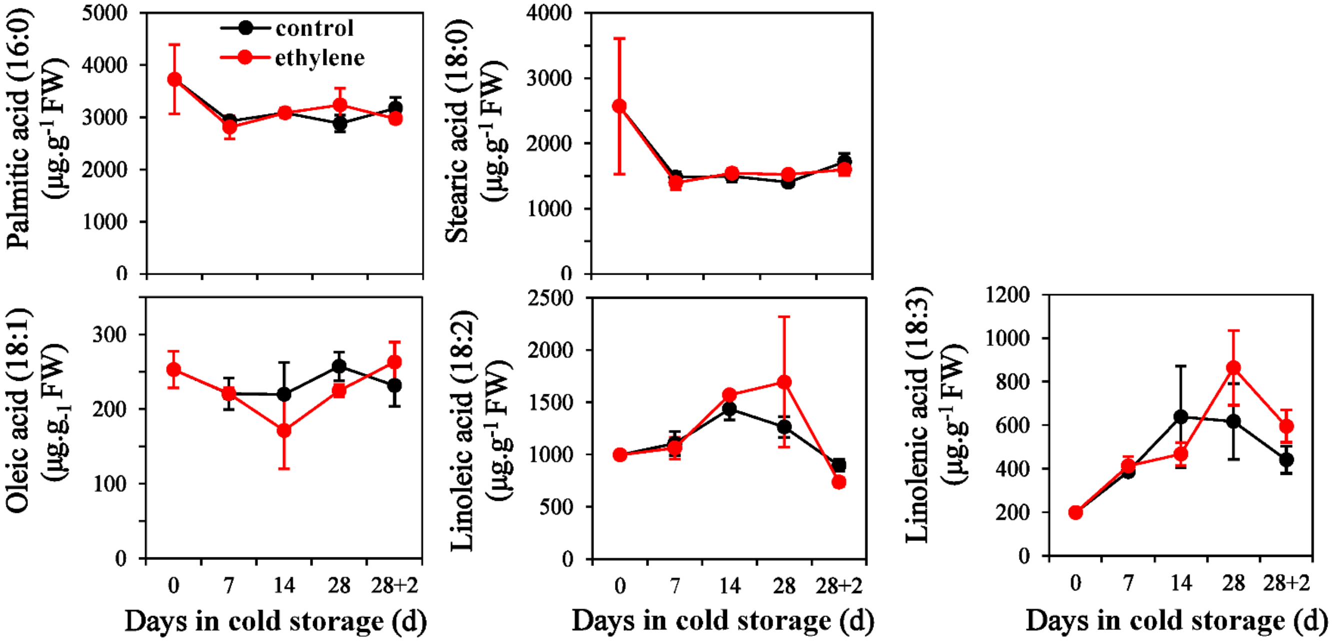

Figure S4. The effect of ethylene on fatty acid levels in peach fruit. Data are presented as mean ±SE from three independent biological replicates.

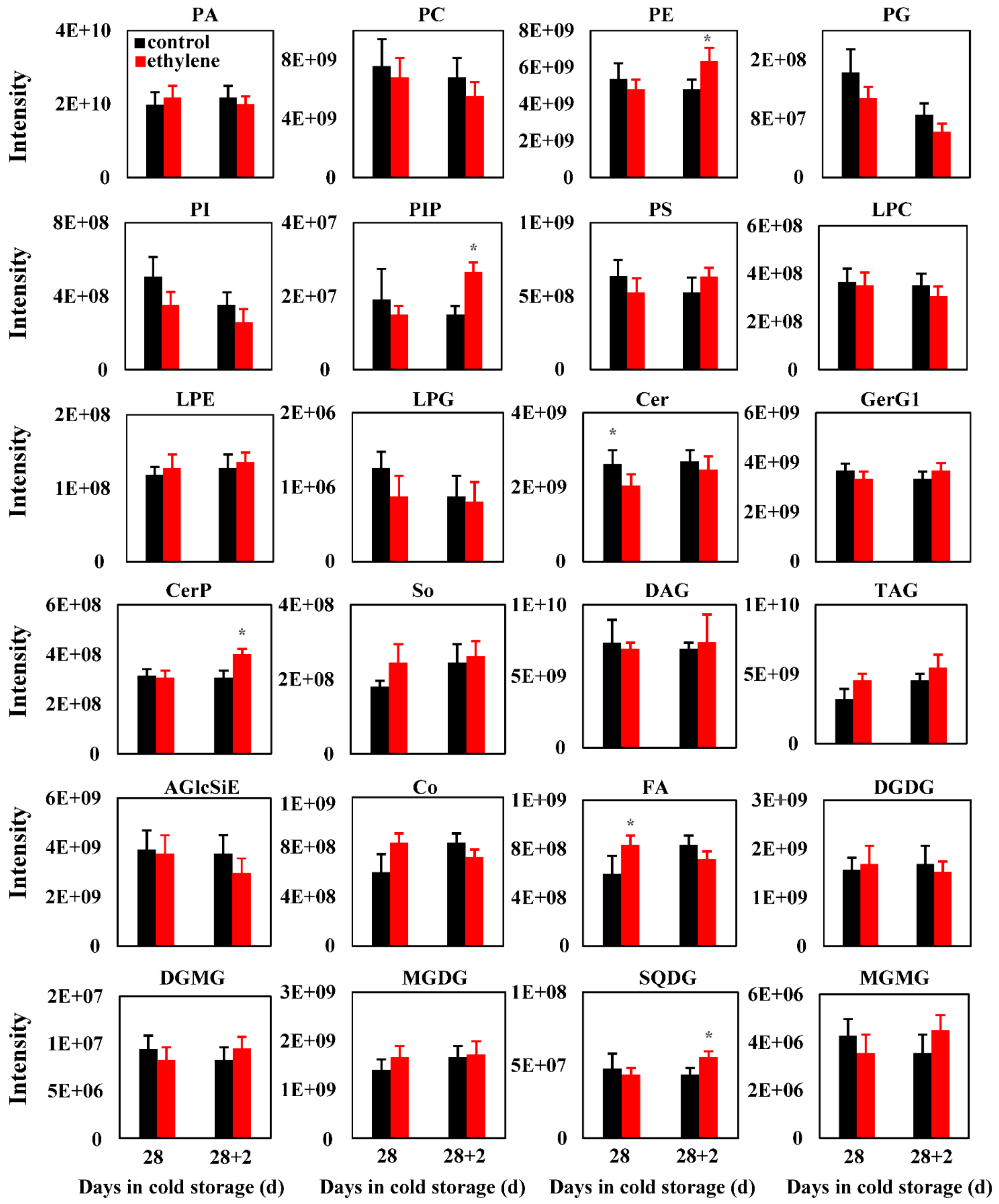

Figure S5. Total content of lipids in control and ethylene-treated fruit at 28 d of cold storage and +2 d of shelf. Data are presented as mean ±SE from four independent biological replicates. Asterisks indicate that mean values are significantly different between the treatments of ethylene and control ($P < 0.05$) according to Duncan's Multiple Range Test. AGlcSiE, AcylGlcSitosterol ester; Cer, ceramide; CerGl, monoglycosylceramide; Cerp, ceramides phosphate; Co, coenzyme; DAG, diacyglyceride; DGDG, digalactosyldiacylglycerol; DGMG, digalactosylmonoacyglycerol; FA, fatty acid; LPC, lysophosphatidylcholine; LPE, lysophosphatidylethanolamine; LPG,lysophosphatidylglycerol; MGDG, monogalactosyldiacylglycerol; MGMG, monogalactosylmonoacylglycerol; PA, phosphatidic acid; PC, phosphatidylcholine; PE, phosphatidylethanolamine; PG, phosphatidylglycerol; PI, phosphatidylinositol; PIP, phosphatidylinositol; PS, phosphatidylserine; So, sphingosine; SQDG, sulfoquinovosyldiacyglycerol; TAG, triacyglyceride.

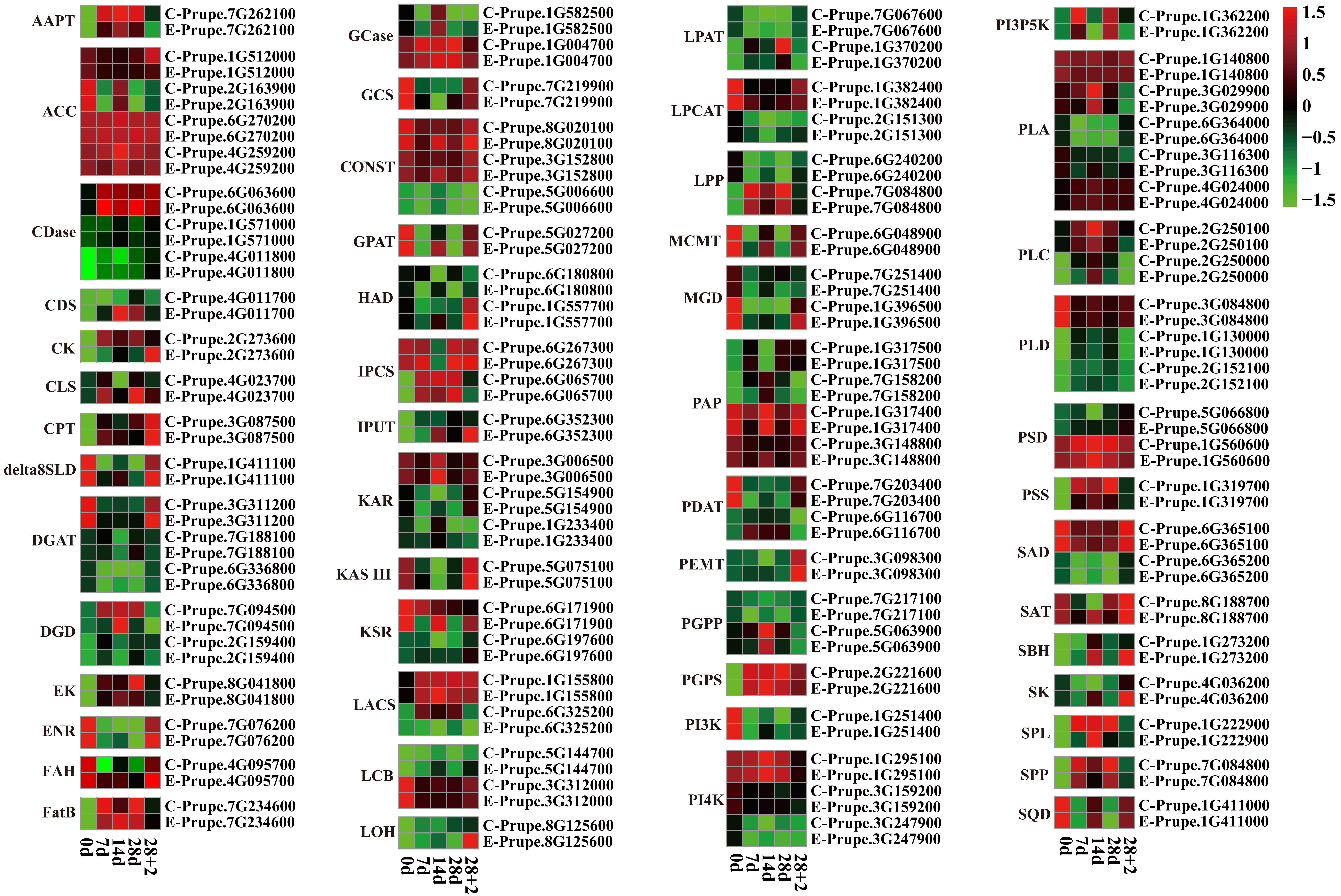

Figure S6. Expression profiling of genes related to lipid metabolism under ethylene treatment and the control. The color gradient, ranging from green, through black, to red represents low, middle and high values of gene expression. The sampling points beginning with letter C or E indicate those belong to control and ethylene treatments, respectively.The abbreviations are defined in the legend to **Fig.** 8.

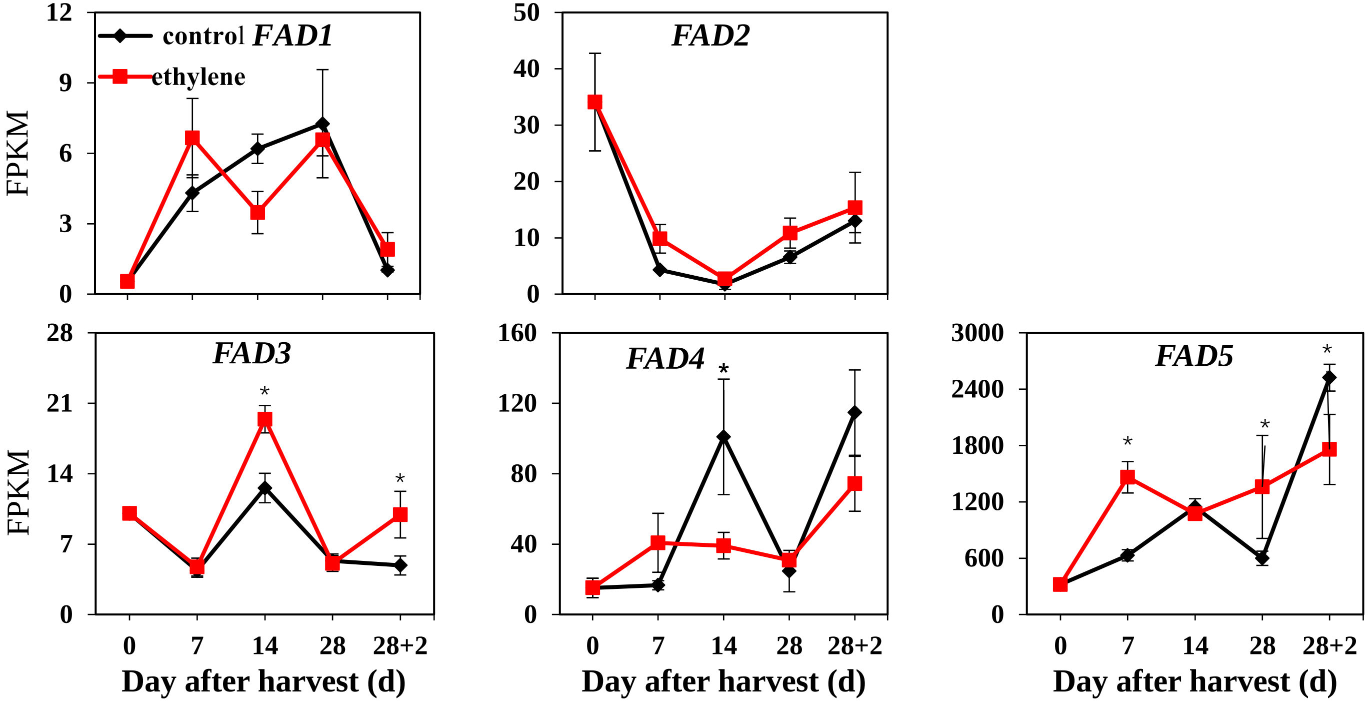

Figure S7. The effect of ethylene on expression of *FADs* in peach fruit. Data are presented as mean ±SE from three independent biological replicates. Asterisks indicate that mean values are significantly different between the treatments of ethylene and control ($P < 0.05$) according to Duncan's Multiple Range Test. FAD, fatty acid desaturase; FPKM, fragments per kilobase of exon per million reads mapped.

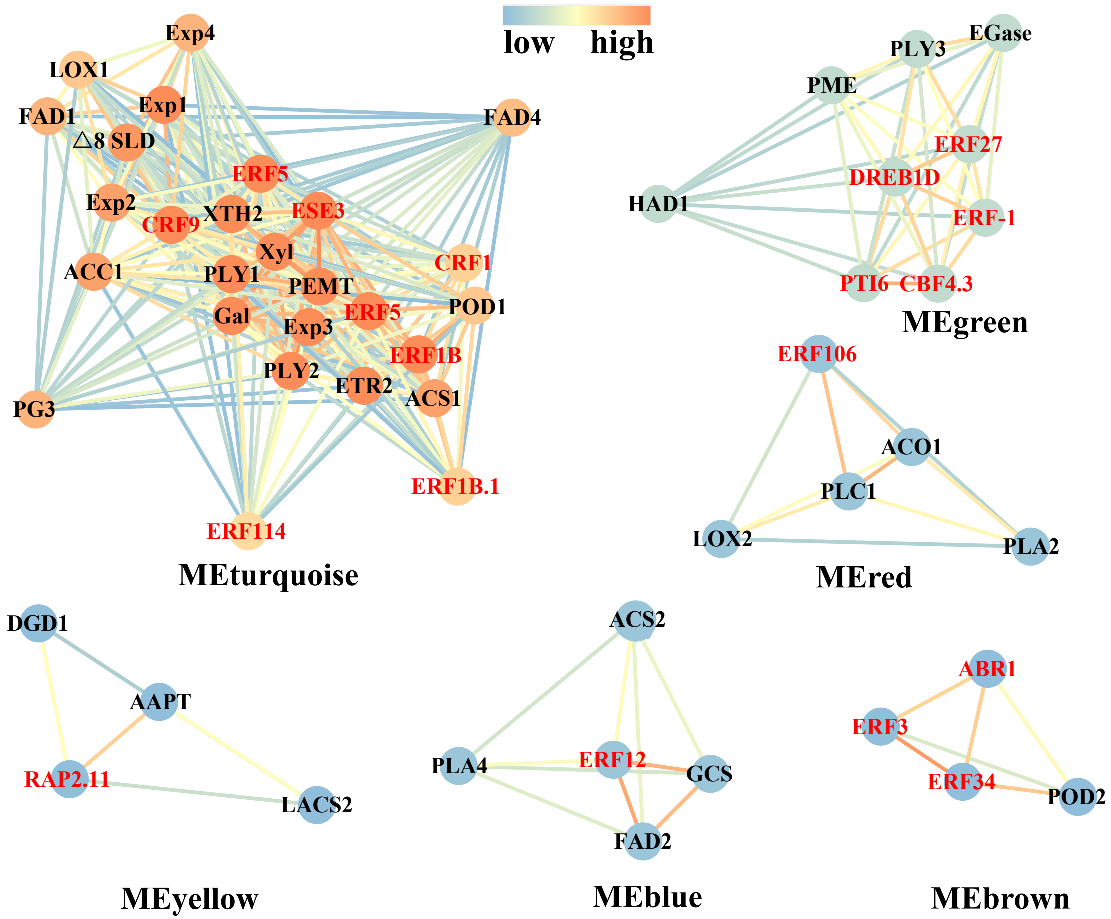

Figure S8. Coexpression network between *ERFs* and genes related to cell wall modification，lipid metabolism, ethylene biosynthesis and internal browning. The circle colour gradient from blue to yellow, indicates increase of degree of node size. The line colour gradient from blue to yellow, indicates increase of weight value of edge size. Genes in red indicate *ERFs*. The abbreviations are defined in the Figures 5, 8 and 10.

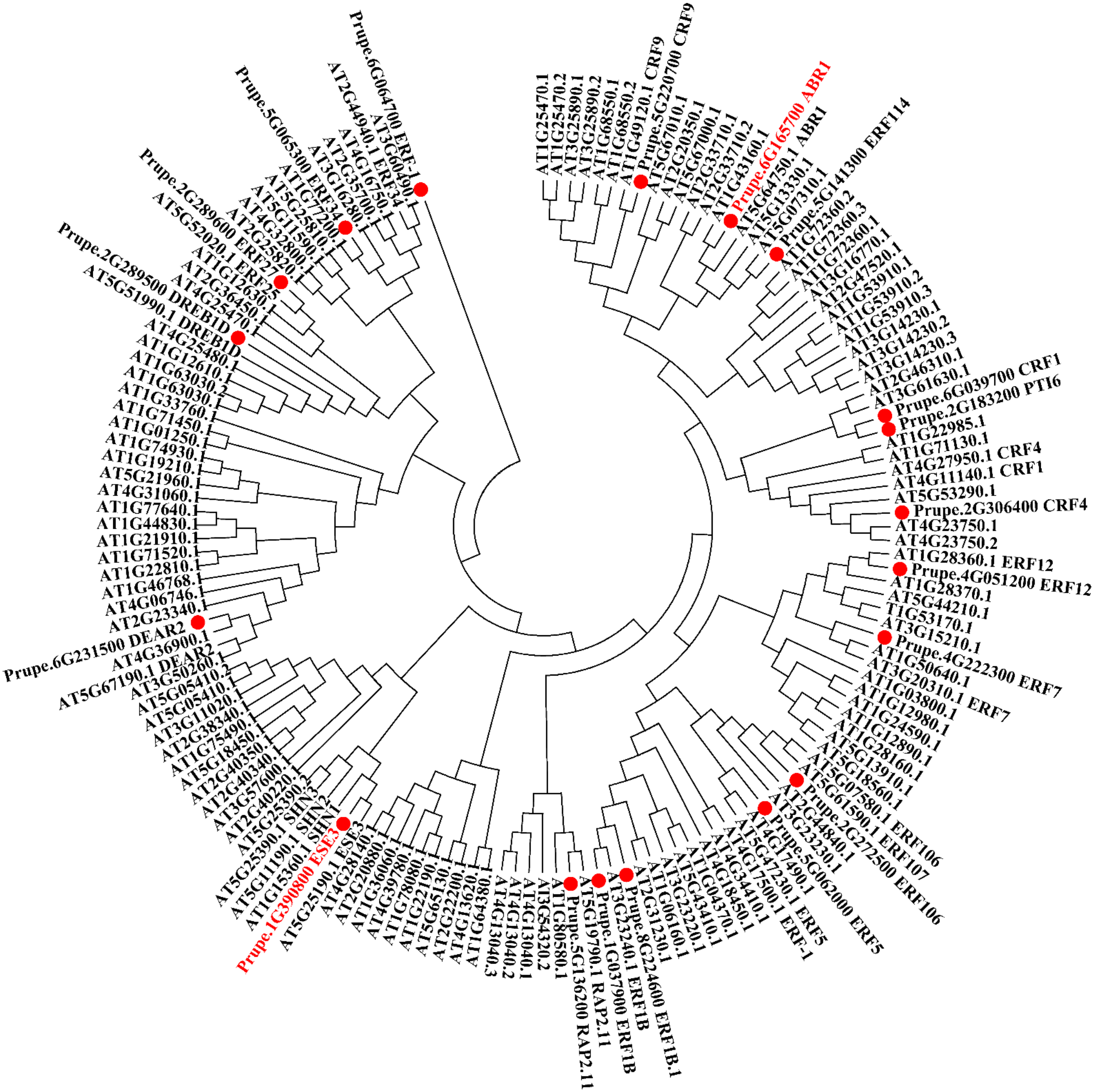

Figure S9. Phylogenetic tree of identified ERFs. Solid red circles indicate peach gene; The remaining genes without label decoration belong to the *Arabidopsis* genome.

Table S1. Primers for real-time quantitative PCR.

Gene	Gene ID	Forward primer (5′ to 3′)	Reverse primer (5′ to 3′)
1-Aminocyclopropane-1-carboxylic acid oxidase (ACO1)	Prupe.3G209900	TCAATGATATGGACTGGGAAAG	GGTTGGGACAAGGAGGGT
1-Aminocyclopropane-1-carboxylic acid synthase (ACS1)	Prupe.2G176900	AGAAAGGCTGTGGCTATG	ATCCCAAGTCTCGGTAAA
Aminoalcoholphosphotransferases (AAPT)	Prupe.7G262100	AAGCAAGCAAGACGGACAA	GCCCATTAACGGCAGGAA
Expansin (Exp1)	Prupe.2G263600	ACTATGGAGGAGGATGGG	AAGAGTGACCGTTGATGG
Galactosidase (Gal)	Prupe.4G278500	TAGGACTTTACAAGGGAGA	TTCAAGGTCAATAGAGGG
Pectate lyase (PLY1)	Prupe.5G161300	AAAGCCAGGGACTCTACGA	TATGCCCAAGCAACATCA
Phospholipase D (PLD1)	Prupe.3G084800	AACCCACGATGAAGAAAC	AGACCACCAACGAAACTC
S-adenosylmethionine synthase (SAMS1)	Prupe.1G107000	AGGAGATTGGTGCTGGTG	ATGGGTTGAGATGGAAGATG
Stearoyl-ACP desaturase (SAD1)	Prupe.6G365100	TCCCATCTCACAACCTCC	TGCCAGCACTTCTCAACA
PpTEF	JQ732180	GGTGTGACGATGAAGAGTGATG	TGAAGGAGAGGGAAGGTGAAAG

Table S2. Quality of reads for 27 RNA-Seq samples. Abbreviations used are as follow: E, ethylene; C, control.

**Sample name**	**Clean reads**	**clean bases**	**Q30 (%)**	**GC content (%)**
0d_1	32,314,227	9,605,293,878	95.47%	46.10%
0d_2	24,165,520	7,181,259,090	95.96%	46.74%
0d_3	27,738,993	8,252,424,248	95.95%	46.47%
C7d_1	23,152,690	6,888,068,990	94.86%	46.74%
C7d_2	25,945,786	7,709,988,648	94.71%	46.60%
C7d_3	24,007,991	7,150,722,178	95.03%	46.86%
E7d_1	23,290,138	6,939,570,860	95.29%	46.55%
E7d_2	23,718,859	7,062,373,082	95.32%	47.41%
E7d_3	20,603,107	6,152,345,128	94.85%	46.24%
C14d_1	23,435,513	7,005,230,142	94.74%	45.99%
C14d_2	25,179,000	7,531,611,508	94.54%	45.90%
C14d_3	29,847,211	8,923,515,962	94.64%	45.85%
E14d_1	30,047,416	8,995,751,812	94.65%	45.78%
E14d_2	28,540,133	8,542,975,198	94.58%	45.78%
E14d_3	27,219,109	8,148,753,194	94.39%	45.78%
C28d_1	27,500,891	8,228,453,284	94.43%	46.27%
C28d_2	29,388,660	8,796,503,218	94.37%	46.47%
C28d_3	28,830,332	8,627,977,358	94.45%	45.75%
E28d_1	29,501,446	8,833,115,880	95.03%	45.72%
E28d_2	27,861,987	8,341,232,474	95.06%	45.64%
E28d_3	25,994,296	7,781,761,052	94.90%	46.01%
C28+2d_1	27,383,585	8,190,062,168	94.98%	46.43%
C28+2d_2	24,662,429	7,378,658,646	95.28%	46.33%
C28+2d_3	26,167,778	7,830,757,930	94.96%	46.02%
E28+2d_1	27,940,093	8,362,073,730	95.00%	45.74%
E28+2d_2	25,969,747	7,772,674,114	95.30%	45.97%
E28+2d_3	26,832,556	8,026,229,600	95.26%	46.12%

Table S3. Mapping statistics for 27 RNA-Seq samples. Abbreviations used are as follow: E, ethylene; C, control.

Sample name	Total reads	Mapped reads	Uniq Mapped Reads	Multiple Map Reads	Reads Map to '+'	Reads Map to '-'
0d_1	64,628,454	59,428,483 (91.95%)	57,605,177 (89.13%)	1,823,306 (2.82%)	29,644,941 (45.87%)	29,710,728 (45.97%)
0d_2	48,331,040	44,192,964 (91.44%)	42,863,254 (88.69%)	1,329,710 (2.75%)	22,043,238 (45.61%)	22,102,296 (45.73%)
0d_3	55,477,986	51,484,678 (92.80%)	50,136,936 (90.37%)	1,347,742 (2.43%)	25,670,760 (46.27%)	25,745,389 (46.41%)
C7d_1	46,305,380	42,375,257 (91.51%)	41,037,722 (88.62%)	1,337,535 (2.89%)	21,144,618 (45.66%)	21,167,947 (45.71%)
C7d_2	51,891,572	47,197,078 (90.95%)	45,796,682 (88.25%)	1,400,396 (2.70%)	23,542,640 (45.37%)	23,577,278 (45.44%)
C7d_3	48,015,982	43,672,720 (90.95%)	42,115,483 (87.71%)	1,557,237 (3.24%)	21,794,809 (45.39%)	21,817,156 (45.44%)
E7d_1	46,580,276	43,876,612 (94.20%)	42,447,251 (91.13%)	1,429,361 (3.07%)	21,881,354 (46.98%)	21,923,300 (47.07%)
E7d_2	47,437,718	42,471,249 (89.53%)	41,242,906 (86.94%)	1,228,343 (2.59%)	21,189,698 (44.67%)	21,214,370 (44.72%)
E7d_3	41,206,214	37,245,607 (90.39%)	35,907,098 (87.14%)	1,338,509 (3.25%)	18,564,826 (45.05%)	18,599,710 (45.14%)
C14d_1	46,871,026	43,128,078 (92.01%)	41,038,330 (87.56%)	2,089,748 (4.46%)	21,540,209 (45.96%)	21,532,586 (45.94%)
C14d_2	50,358,000	46,304,562 (91.95%)	43,939,692 (87.25%)	2,364,870 (4.70%)	23,138,832 (45.95%)	23,118,178 (45.91%)
C14d_3	59,694,422	55,877,653 (93.61%)	52,386,622 (87.76%)	3,491,031 (5.85%)	27,908,143 (46.75%)	27,889,882 (46.72%)
E14d_1	60,094,832	56,271,989 (93.64%)	53,686,668 (89.34%)	2,585,321 (4.30%)	28,027,082 (46.64%)	28,080,290 (46.73%)
E14d_2	57,080,266	53,619,522 (93.94%)	51,188,246 (89.68%)	2,431,276 (4.26%)	26,732,928 (46.83%)	26,765,097 (46.89%)
E14d_3	54,438,218	50,772,527 (93.27%)	48,107,950 (88.37%)	2,664,577 (4.89%)	25,314,285 (46.50%)	25,338,363 (46.55%)
C28d_1	55,001,782	50,922,010 (92.58%)	49,037,323 (89.16%)	1,884,687 (3.43%)	25,417,178 (46.21%)	25,444,459 (46.26%)
C28d_2	58,777,320	53,353,330 (90.77%)	51,542,498 (87.69%)	1,810,832 (3.08%)	26,645,930 (45.33%)	26,641,422 (45.33%)
C28d_3	57,660,664	52,890,663 (91.73%)	50,971,149 (88.40%)	1,919,514 (3.33%)	26,410,153 (45.80%)	26,406,432 (45.80%)
E28d_1	59,002,892	55,976,943 (94.87%)	53,969,107 (91.47%)	2,007,836 (3.40%)	27,926,237 (47.33%)	27,967,544 (47.40%)
E28d_2	55,723,974	52,526,065 (94.26%)	50,727,059 (91.03%)	1,799,006 (3.23%)	26,199,414 (47.02%)	26,245,457 (47.10%)
E28d_3	51,988,592	48,066,228 (92.46%)	46,293,987 (89.05%)	1,772,241 (3.41%)	23,967,944 (46.10%)	24,007,692 (46.18%)
C28+2d_1	54,767,170	51,286,359 (93.64%)	49,928,584 (91.17%)	1,357,775 (2.48%)	25,600,421 (46.74%)	25,633,528 (46.80%)
C28+2d_2	49,324,858	44,605,633 (90.43%)	43,536,696 (88.27%)	1,068,937 (2.17%)	22,254,993 (45.12%)	22,281,680 (45.17%)
C28+2d_3	52,335,556	48,148,874 (92.00%)	47,128,873 (90.05%)	1,020,001 (1.95%)	24,043,526 (45.94%)	24,059,110 (45.97%)
E28+2d_1	55,880,186	52,276,719 (93.55%)	51,204,802 (91.63%)	1,071,917 (1.92%)	26,082,138 (46.68%)	26,101,674 (46.71%)
E28+2d_2	51,939,494	47,910,646 (92.24%)	46,536,356 (89.60%)	1,374,290 (2.65%)	23,844,012 (45.91%)	23,899,069 (46.01%)
E28+2d_3	53,665,112	49,847,373 (92.89%)	48,686,432 (90.72%)	1,160,941 (2.16%)	24,870,661 (46.34%)	24,901,330 (46.40%)

Table S4. Correlation matrix between 27 RNA-Seq samples. Abbreviations used are as follow: E, ethylene; C, control.

#Sample	0d_1	0d_2	0d_3	C7d_1	C7d_2	C7d_3	E7d_1	E7d_2	E7d_3	C14d_1	C14d_2	C14d_3	E14d_1	E14d_2	E14d_3
0d_1	1	0.9312	0.9124	0.4507	0.4555	0.4114	0.3325	0.5558	0.3907	0.6094	0.5825	0.6322	0.4394	0.5991	0.5861
0d_2	0.9312	1	0.9277	0.479	0.4847	0.433	0.349	0.5978	0.4074	0.6327	0.6065	0.6419	0.4305	0.5906	0.5886
0d_3	0.9124	0.9277	1	0.4191	0.4143	0.3788	0.3178	0.558	0.3855	0.6017	0.5559	0.6036	0.4263	0.5968	0.5766
C7d_1	0.4507	0.479	0.4191	1	0.9762	0.9766	0.825	0.8417	0.7717	0.8795	0.8871	0.8763	0.585	0.6934	0.7446
C7d_2	0.4555	0.4847	0.4143	0.9762	1	0.9682	0.7875	0.8191	0.7394	0.8855	0.9027	0.8739	0.5851	0.6831	0.7321
C7d_3	0.4114	0.433	0.3788	0.9766	0.9682	1	0.8424	0.8147	0.7906	0.8539	0.8647	0.8496	0.5747	0.6801	0.734
E7d_1	0.3325	0.349	0.3178	0.825	0.7875	0.8424	1	0.8249	0.8962	0.7169	0.7078	0.7137	0.6795	0.7141	0.7648
E7d_2	0.5558	0.5978	0.558	0.8417	0.8191	0.8147	0.8249	1	0.8118	0.8138	0.7981	0.805	0.6324	0.7212	0.7613
E7d_3	0.3907	0.4074	0.3855	0.7717	0.7394	0.7906	0.8962	0.8118	1	0.7229	0.6839	0.7312	0.6743	0.7057	0.7559
C14d_1	0.6094	0.6327	0.6017	0.8795	0.8855	0.8539	0.7169	0.8138	0.7229	1	0.9697	0.9765	0.691	0.8352	0.8651
C14d_2	0.5825	0.6065	0.5559	0.8871	0.9027	0.8647	0.7078	0.7981	0.6839	0.9697	1	0.9711	0.6657	0.7901	0.8352
C14d_3	0.6322	0.6419	0.6036	0.8763	0.8739	0.8496	0.7137	0.805	0.7312	0.9765	0.9711	1	0.6941	0.8326	0.8711
E14d_1	0.4394	0.4305	0.4263	0.585	0.5851	0.5747	0.6795	0.6324	0.6743	0.691	0.6657	0.6941	1	0.8917	0.8863
E14d_2	0.5991	0.5906	0.5968	0.6934	0.6831	0.6801	0.7141	0.7212	0.7057	0.8352	0.7901	0.8326	0.8917	1	0.981
E14d_3	0.5861	0.5886	0.5766	0.7446	0.7321	0.734	0.7648	0.7613	0.7559	0.8651	0.8352	0.8711	0.8863	0.981	1
C28d_1	0.4272	0.4448	0.3993	0.9288	0.9306	0.9582	0.8361	0.7851	0.7363	0.8415	0.8577	0.8322	0.583	0.6972	0.7467
C28d_2	0.3643	0.3797	0.3347	0.9526	0.9464	0.9716	0.849	0.8	0.7689	0.8446	0.854	0.8307	0.5863	0.6897	0.7399
C28d_3	0.3899	0.4096	0.3536	0.9587	0.9597	0.9665	0.8499	0.804	0.762	0.8653	0.8843	0.8514	0.6102	0.7013	0.7512
E28d_1	0.295	0.3018	0.2684	0.7001	0.6606	0.7151	0.9406	0.7512	0.8822	0.6229	0.5976	0.6258	0.7382	0.7205	0.761
E28d_2	0.3603	0.3824	0.3243	0.7688	0.7398	0.7753	0.9163	0.8816	0.9188	0.7073	0.6852	0.7014	0.7194	0.7296	0.772
E28d_3	0.4065	0.4248	0.3905	0.8298	0.7935	0.8485	0.9413	0.8264	0.9721	0.7689	0.7375	0.776	0.706	0.7463	0.7993
C28+2d_1	0.4172	0.4426	0.3982	0.8136	0.8169	0.8188	0.8089	0.838	0.6919	0.7199	0.7404	0.7032	0.5655	0.6341	0.6717
C28+2d_2	0.4061	0.4447	0.3915	0.6695	0.6942	0.6668	0.687	0.7947	0.6188	0.6145	0.6213	0.5852	0.5564	0.5815	0.5977
C28+2d_3	0.4184	0.4549	0.4204	0.6569	0.6623	0.6418	0.6585	0.8116	0.5765	0.6004	0.6136	0.574	0.4708	0.5299	0.5491
E28+2d_1	0.3815	0.4012	0.4066	0.4408	0.4482	0.4289	0.5165	0.6597	0.4422	0.4202	0.4159	0.3819	0.4486	0.4673	0.4505
E28+2d_2	0.3245	0.3606	0.3376	0.4888	0.4854	0.4752	0.5937	0.642	0.5317	0.4532	0.4411	0.4227	0.6232	0.5621	0.5637
E28+2d_3	0.4064	0.4553	0.4188	0.6141	0.6165	0.612	0.7046	0.8191	0.7172	0.5841	0.5662	0.5513	0.5821	0.5934	0.6069

#Sample	C28d_1	C28d_2	C28d_3	E28d_1	E28d_2	E28d_3	C28+2d_1	C28+2d_2	C28+2d_3	E28+2d_1	E28+2d_2	E28+2d_3
0d_1	0.4272	0.3643	0.3899	0.295	0.3603	0.4065	0.4172	0.4061	0.4184	0.3815	0.3245	0.4064
0d_2	0.4448	0.3797	0.4096	0.3018	0.3824	0.4248	0.4426	0.4447	0.4549	0.4012	0.3606	0.4553
0d_3	0.3993	0.3347	0.3536	0.2684	0.3243	0.3905	0.3982	0.3915	0.4204	0.4066	0.3376	0.4188
C7d_1	0.9288	0.9526	0.9587	0.7001	0.7688	0.8298	0.8136	0.6695	0.6569	0.4408	0.4888	0.6141
C7d_2	0.9306	0.9464	0.9597	0.6606	0.7398	0.7935	0.8169	0.6942	0.6623	0.4482	0.4854	0.6165
C7d_3	0.9582	0.9716	0.9665	0.7151	0.7753	0.8485	0.8188	0.6668	0.6418	0.4289	0.4752	0.612
E7d_1	0.8361	0.849	0.8499	0.9406	0.9163	0.9413	0.8089	0.687	0.6585	0.5165	0.5937	0.7046
E7d_2	0.7851	0.8	0.804	0.7512	0.8816	0.8264	0.838	0.7947	0.8116	0.6597	0.642	0.8191
E7d_3	0.7363	0.7689	0.762	0.8822	0.9188	0.9721	0.6919	0.6188	0.5765	0.4422	0.5317	0.7172
C14d_1	0.8415	0.8446	0.8653	0.6229	0.7073	0.7689	0.7199	0.6145	0.6004	0.4202	0.4532	0.5841
C14d_2	0.8577	0.854	0.8843	0.5976	0.6852	0.7375	0.7404	0.6213	0.6136	0.4159	0.4411	0.5662
C14d_3	0.8322	0.8307	0.8514	0.6258	0.7014	0.776	0.7032	0.5852	0.574	0.3819	0.4227	0.5513
E14d_1	0.583	0.5863	0.6102	0.7382	0.7194	0.706	0.5655	0.5564	0.4708	0.4486	0.6232	0.5821
E14d_2	0.6972	0.6897	0.7013	0.7205	0.7296	0.7463	0.6341	0.5815	0.5299	0.4673	0.5621	0.5934
E14d_3	0.7467	0.7399	0.7512	0.761	0.772	0.7993	0.6717	0.5977	0.5491	0.4505	0.5637	0.6069
C28d_1	1	0.9666	0.9604	0.7176	0.7436	0.8217	0.8248	0.6536	0.6285	0.4421	0.501	0.5942
C28d_2	0.9666	1	0.9743	0.7301	0.7742	0.842	0.8446	0.6721	0.652	0.4513	0.5061	0.6097
C28d_3	0.9604	0.9743	1	0.7374	0.7798	0.841	0.8393	0.68	0.6631	0.457	0.5074	0.616
E28d_1	0.7176	0.7301	0.7374	1	0.9175	0.9186	0.7213	0.624	0.5774	0.4744	0.5896	0.6832
E28d_2	0.7436	0.7742	0.7798	0.9175	1	0.9177	0.7981	0.7496	0.7134	0.5983	0.6579	0.8177
E28d_3	0.8217	0.842	0.841	0.9186	0.9177	1	0.7571	0.6417	0.6036	0.4528	0.5535	0.6983
C28+2d_1	0.8248	0.8446	0.8393	0.7213	0.7981	0.7571	1	0.8812	0.892	0.7375	0.7024	0.8173
C28+2d_2	0.6536	0.6721	0.68	0.624	0.7496	0.6417	0.8812	1	0.9317	0.8748	0.8323	0.9129
C28+2d_3	0.6285	0.652	0.6631	0.5774	0.7134	0.6036	0.892	0.9317	1	0.8705	0.7496	0.8924
E28+2d_1	0.4421	0.4513	0.457	0.4744	0.5983	0.4528	0.7375	0.8748	0.8705	1	0.857	0.8521
E28+2d_2	0.501	0.5061	0.5074	0.5896	0.6579	0.5535	0.7024	0.8323	0.7496	0.857	1	0.8272
E28+2d_3	0.5942	0.6097	0.616	0.6832	0.8177	0.6983	0.8173	0.9129	0.8924	0.8521	0.8272	1

Table S5. List of names and IDs of genes involved in ethylene biosynthesis and signaling, cell wall modification, and browning. The abbreviations used are listed in the legend to Fig. 3.

Gene name	Gene ID	Gene name	Gene ID
SAMS1	Prupe.1G107000	PLY3	Prupe.2G206100
SAMS2	Prupe.7G128500	PME	Prupe.7G192800
ACS1	Prupe.2G176900	Egase	Prupe.5G131300
ACS2	Prupe.5G083500	XyL	Prupe.1G309900
ACO1	Prupe.3G209900	Exp1	Prupe.2G263600
ACO2	Prupe.4G013800	Exp2	Prupe.1G276700
ETR1	Prupe.1G556000	Exp3	Prupe.6G075100
ETR2	Prupe.1G034300	Exp4	Prupe.8G174500
ERS1	Prupe.8G265200	Gal	Prupe.4G278500
EIN2	Prupe.6G235600	GS	Prupe.1G289600
EIN3	Prupe.2G058400	XTH1	Prupe.8G214900
EIN4	Prupe.6G348000	XTH2	Prupe.1G166300
CTR1	Prupe.7G117700	PPO1	Prupe.4G042100
EIL3	Prupe.6G018200	PPO2	Prupe.4G042000
PG3	Prupe.2G301000	POD1	Prupe.8G038900
PG4	Prupe.7G195000	POD2	Prupe.4G021100
PLY1	Prupe.5G161300	LOX1	Prupe.4G047800
PLY2	Prupe.1G060900	LOX2	Prupe.6G324100

Table S6. The results of multiple comparison of lipid gene expression after one-way ANOVA at 5% significance level. Red arrows indicate significantly higher level in ethylene compared with control, and green arrows mean significantly lower level. Significantly higher level in one treatment were defined as at least two time points with significantly higher level while without any time point showing significantly lower level, and allowing presence of time points with no significant difference. The total abundance of transcripts was employed for genes with multiple differential expressed members. The abbreviations used are listed in the legend to Fig. 8.

Gene	7d	14d	28d	28+2d	General
Δ8SLD	—	—	—	↑	—
AAPT	↓	↓	↓	—	↓
ACC1	—	—	—	↓	—
ACC2	—	—	—	—	—
ACC3	—	—	—	—	—
ACC4	↓	↓	↓	—	↓
ACC total	—	↓	—	—	—
CDase1	↑	—	↑	—	↑
CDase2	—	—	—	—	—
CDase3	—	↑	—	—	—
CDase total	↑	—	↑	—	↑
CDS	—	↑	—	—	—
CK	—	—	—	—	—
CLS	—	↓	—	↓	[illegible]
CPT	—	—	—	—	—
DGAT1	—	—	—	↑	—
DGAT2	—	—	—	—	—
DGAT3	—	—	—	—	—
DGAT total	—	—	—	↑	—
DGD1	↓	—	↓	—	↓
DGD2	—	—	—	—	—
DGD total	↓	—	↓	—	↓
DGK	—	↑	—	—	—
EK	—	—	↓	—	—
ENR	—	—	—	↑	—
FAH	↑	—	—	↑	↑
FatB	—	↑	—	—	—

Gene	7d	14d	28d	28+2d	General
GCase1	—	—	—	—	—
GCase2	—	—	—	—	—
GCase total	—	—	—	—	—
GCS	—	—	↑	—	—
GONST1	—	↑	—	↑	↑
GONST2	—	↑	—	—	—
GONST3	—	—	—	—	—
GONST total	—	↑	—	↑	↑
GPAT	—	↑	—	—	—
HAD1	↓	↑	↓	—	—
HAD2	—	↑	—	↑	↑
HAD total	—	↑	—	↑	↑
IPCS1	—	—	—	—	—
IPCS2	—	—	—	—	—
IPCS total	—	—	—	—	—
IPUT	—	—	—	—	—
KAR1	—	↑	—	—	—
KAR2	—	↑	—	—	—
KAR3	—	—	—	—	—
KAR total	—	↑	—	—	—
KAS III	—	—	—	—	—
KSR1	↓	↑	↓	↑	—
KSR2	—	↑	—	—	—
KSR total	↓	↑	—	↑	—
lACS1	—	↑	—	—	—
LACS2	↓	↓	↓	—	↓
LACS total	↓	↓	↓	—	↓
LCB1	—	—	—	↑	—
LCB2	—	↑	—	↑	↑
LCB3	—	—	—	—	—
LCB total	—	—	—	↑	—
LOH	—	—	—	↑	—

Gene	7d	14d	28d	28+2d	General
LPAT1	—	↑	—	—	—
LPAT2	—	—	↓	—	—
LPAT total	—	—	↓	—	—
LPCAT1	↑	—	↑	—	↑
LPCAT2	—	—	↑	↑	↑
LPCAT total	↑	—	↑	—	↑
LPP1	—	↑	—	↑	↑
LPP2	—	—	—	↑	—
LPP total	—	↑	—	↑	↑
MCMT	—	—	—	—	—
MGD1	—	—	↓	—	—
MGD2	—	—	—	—	—
MGD total	—	—	—	—	—
PAP1	—	—	—	—	—
PAP2	↓	—	↓	—	↓
PAP3	—	—	—	—	—
PAP4	—	↑	—	↑	↑
PAP total	—	—	—	—	—
PDAT1	—	—	—	—	—
PDAT2	↑	↑	↑	—	↑
PDAT total	↑	↑	—	—	∣
PEMT	—	—	—	↑	—
PGPP1	↓	—	—	—	—
PGPP2	↓	↓	↓	—	↓
PGPP total	↓	↓	↓	—	↓
PGPS	—	—	—	—	—
PI3K	—	↑	↑	—	↑
PI3PK5	↓	↓	—	↓	↓
PI4K1	—	—	—	—	—
PI4K2	—	—	—	—	—
PI4K3	—	↑	—	—	—
PI4K total	—	—	—	—	—

Gene	7d	14d	28d	28+2d	General
PLA1	—	↓	—	↓	↓
PLA2	—	↓	—	—	—
PLA3	—	—	—	—	—
PLA4	—	↑	—	↑	↑
PLA5	↑	—	↑	—	↑
PLA total	—	↓	—	—	—
PLC1	—	↓	—	—	—
PLC2	↓	↑	↓	—	—
PLC total	—	↓	—	—	—
PLD1	—	—	—	—	—
PLD2	—	—	—	—	—
PLD3	—	—	—	—	—
PLD total	—	—	—	—	—
PSD1	—	↑	—	—	—
PSD2	—	—	—	—	—
PSD total	—	—	—	—	—
PSS	↓	—	↓	—	↓
SAD1	—	—	—	—	—
SAD2	—	—	—	—	—
SAD total	—	—	—	—	—
SAT	—	↑	—	—	—
SBH	—	—	—	↑	—
SK	—	—	—	—	—
SPL	↓	—	↓	—	↓
SPP	—	—	↓	—	—
SQD	—	—	—	—	—

Table S7. Expression profiles (FPKM) of differentially expressed *ERFs*. Abbreviations used are as follow: E, ethylene, C, control.

Module	Gene Name	GeneID	0d	C7d	E7d	C14d	E14d	C28d	E28d	C28+2d	E28+2d
turquoise	ESE3	Prupe.1G390800	0.8128	0.0061	1.6590	0.0000	0.1385	0.1552	1.1886	22.6974	45.1718
	ERF1B	Prupe.1G037900	0.0620	0.4090	1.0302	0.1760	1.0700	0.9801	2.0295	1.6328	5.5516
	CRF1	Prupe.6G039700	1.8397	1.0676	4.0309	0.1059	0.1842	0.6959	6.3033	1.7221	8.2583
	ERF1B.1	Prupe.8G224600	0.2130	0.4269	1.3366	2.0432	2.2222	1.1838	3.5954	1.5377	4.3718
	CRF9	Prupe.5G220700	5.1227	1.7893	2.2944	0.3792	1.3738	1.5464	2.5926	5.0989	5.4680
	ERF114	Prupe.5G141300	0.0563	0.0944	0.7045	0.0392	0.5115	0.4625	1.2071	1.6159	2.7457
	ERF5	Prupe.5G062000	34.2848	54.0139	34.8534	58.8172	27.6126	45.9439	34.8540	13.4144	16.0334
others	ERF7	Prupe.4G222300	49.7214	203.6252	109.9984	70.6034	28.5607	128.7197	82.1091	76.3180	40.8483
	ERF106	Prupe.2G272500	12.9350	17.8615	8.0352	37.6790	35.1910	13.0418	9.2613	6.0954	14.2577
	ABR1	Prupe.6G165700	0.4872	51.3363	37.4601	23.3433	17.1363	60.3310	41.4946	11.6663	3.6319
	RAP2.11	Prupe.5G136200	0.1113	1.6775	0.5074	0.7371	1.3987	0.8118	0.6293	0.1997	0.1855
	ERF34	Prupe.5G065300	0.3435	3.4844	1.5462	0.9151	0.3307	3.2431	1.1374	0.4909	0.1488
	ERF-1	Prupe.6G064700	70.9426	32.7800	150.1019	3.1852	4.3251	40.2304	158.4721	74.8273	53.3249
	DREB1D	Prupe.2G289500	1.0115	4.3762	15.0481	0.2542	0.4691	2.3667	17.6989	3.5435	11.3061
	ERF12	Prupe.4G051200	20.2165	1.1254	3.7722	0.4262	0.6472	1.4691	3.1325	7.2129	7.5394
	DEAR2	Prupe.6G231500	2.3295	2.0213	2.3486	1.1817	3.2475	1.7189	1.5986	2.3922	2.5827
	CRF4	Prupe.2G306400	0.1464	4.0169	9.0632	0.2472	0.3458	4.4312	13.6924	2.0082	2.4144
	ERF27	Prupe.2G289600	0.2174	0.2654	1.3218	0.1122	0.0888	0.3043	1.9182	0.5089	1.3411
	PTI6	Prupe.2G183200	0.0217	0.8545	3.3690	0.0087	0.0454	1.5302	4.4650	0.2622	0.2365
